# High-Frequency Microfluidic Fractionation for Compound-Resolved Bioactivity-Based Metabolomics

**DOI:** 10.1021/acs.analchem.5c04612

**Published:** 2025-10-25

**Authors:** Christian Geibel, Julian Schubert, Simon B. Knoblauch, Albert Hernandez, Leonardo Boldt, Dana C. Schneider, Stilianos Papadopoulos Lambidis, Giovanni Andrea Vitale, Jakub Fleischer, Manuela Haussmann, Harald Gross, Mingxun Wang, Heike Brötz-Oesterhelt, Daniel Petras

**Affiliations:** † Department of Microbial Bioactive Compounds, Interfaculty Institute of Microbiology and Infection Medicine (IMIT), 9188 University of Tübingen, 72076 Tübingen, Germany; ‡ Cluster of Excellence Controlling Microbes to Fight Infections (CMFI), University of Tübingen, 72076 Tübingen, Germany; § German Center for Infection Research (DZIF), Partner Site Tübingen, 72076 Tübingen, Germany; ∥ Department of Microbiome Science, Max Planck Institute for Biology, 72076 Tübingen, Germany; ⊥ 27255 University of Innsbruck, 6020 Innsbruck, Austria; # Pharmaceutical Institute, Department of Pharmaceutical Biology, University of Tübingen, 72076 Tübingen, Germany; ∇ Department of Computer Science and Engineering, 8790 University of California Riverside, Riverside, California 92521, United States; ○ Department of Biochemistry, University of California Riverside, Riverside, California 72521, United States

## Abstract

Specialized metabolites represent a prolific source of potential drug candidates. However, the process from detecting bioactivity in a crude metabolite extract to unambiguously identifying the active agent is a tedious and expensive endeavor. Speeding up this procedure is crucial, as new drugs, such as antibiotics, are urgently needed. Furthermore, the systematic functional assessment of complex metabolome samples represents a key bottleneck in nontargeted metabolomics, which once solved, holds the potential to fundamentally advance our systematic understanding of biology. To tackle this central bioanalytical challenge, we developed a compound-resolved bioactivity-based metabolomics workflow that combines nontargeted liquid chromatography tandem mass spectrometry (LC-MS/MS), high frequency fractionation on microfluidic devices and subsequent readout with luminescent bioreporter strains. Central for this workflow is a custom high-speed (∼1 Hz frequency) fractionation device that spots the mobile phase onto a microfluidic paper-analytical device (μPAD) in parallel to MS/MS data acquisition. Subsequently, the μPAD can be overlaid with a bioreporter strain, which displays cellular stress by expressing luciferase. The luminescence signal can then be correlated to MS signals through their chromatographic profiles. We evaluated five different luciferase-expressing bioreporter strains which provide information about different antibacterial modes of action, and tested the workflow with different antibiotic standards and mixtures thereof, as well as crude extracts from the known antibiotic producer *Saccharopolyspora erythraea*. Our results demonstrated high sensitivity (up to 1 ng/spot, depending on compound and bioreporter) and the rapid identification of multiple antimicrobial compounds out of crude extracts, highlighting the practicality and high-throughput capability of this compound-resolved bioactivity-based metabolomics approach.

## Graphical abstract

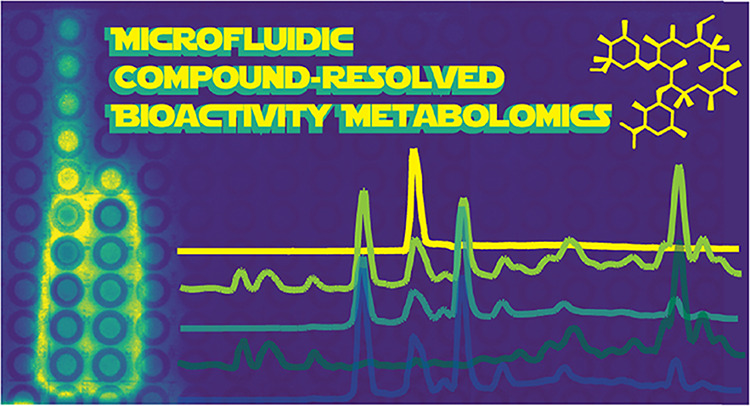

## Introduction

Specialized metabolites (often referred to as natural products) from plants, bacteria, fungi, and other organisms have long served as a rich source of bioactive compounds. Natural products have inspired drug development for decades, either through direct use or by mimicking and optimizing their structures to improve potency, stability, or target binding.^1–3^ Given nature’s vast chemical diversity, it is believed that many specialized metabolites remain undiscovered.^4^ The urgent need for new pharmaceutical lead structures is particularly evident in the case of antibiotics, where nearly 5 million deaths associated with antibiotic resistance were reported in 2019 alone.^5^ At the same time, the ongoing loss of biodiversity threatens to erase natural sources of potential new drugs before they are discovered.^6^ To counter this, rapid screening of natural extracts for bioactive metabolites is of high importance for future drug discovery and fundamental understanding of biological mechanisms. Understanding the structure and pharmacological mode of action of promising compounds allows for their synthesis, optimization and potential medical use.

Despite recent reductions in natural product programs in the pharmaceutical industry, natural products remain a prime resource for drug discovery, although most low-hanging fruit seem to have already been picked, i.e., the potent, highly produced and easy to purify bioactive agents from lab strains. However, this trend is largely driven by challenges in accessing uncultivable taxa, and to fully trigger the biosynthetic potential of cultivable ones.^7–9^ A recent shift in focus includes screening whole microbial communities and field-grown organisms, which are more likely to produce defensive chemicals than laboratory grown strains.^10^ Such approaches hold promise but they require concomitant technology development to increase resolution, sensitivity and throughput. The traditional process of finding new active metabolites is tedious as well as time- and resource-intensive, consisting of an extract being fractionated, and each fraction containing a putative novel and interesting bioactive agent to be subfractionated until the molecule/s responsible for the activity is/are isolated in sufficient amount/s and purity for structure elucidation and biological assessment. This process is usually performed on semipreparative HPLC systems without concomitantly recording mass spectra or obtaining any other information on the fraction, except for the retention time and UV spectra. The antibacterial activity of fractions is usually assessed by inhibition zone tests. This assay is comparably insensitive as it relies on bacterial growth inhibition and does not detect the stress responses experienced by the cells at lower, sublethal concentrations. In this traditional approach, the molecule of interest is identified only at the end of this time-consuming process, often leading to rediscovery of already known bioactive compounds.

Over the last decades, significant progress was made in the high-throughput screening of bioactive compounds. As affinity is a prerequisite to bioactivity, many experimental setups focus on the affinity between natural products and a target. Here, mainly native MS and affinity-selection MS methods have been used.^8^ In native metabolomics, the eluting metabolites from an HPLC system are tested for their affinity against purified proteins, which are infused postcolumn.^11,12^ Affinity-selection MS uses an incubation step as the first step. After incubation, nonbinders are removed either by size-exclusion chromatography or pulsed ultrafiltration. Subsequently, the binder is released by denaturing the protein. This binder can then be detected in an LC-MS run.^13^ Building up from traditional bioactivity-guided natural product discovery,^14^ offline methods for the determination of bioactivity have been widely used. Here, bioactive extracts are separated by LC and fractionated into a deep-well plate. A mass spectrum is then recorded in parallel by infusing a small amount of the eluate in the MS. The results from the offline biotesting are overlaid to the mass spectrum to find possible bioactive compounds.^15^ In this way, e.g., antiprotozoic compounds were identified.^16,17^ While offline approaches are inherently lower throughput, new workflows have been developed to screen extracts in an online manner, e.g., against acetylcholine binding protein (AChBP).^15^ Simultaneously to micro fractionation, mass spectra were recorded via a postcolumn T-piece and infusing a small amount of the eluate into the MS. Other sample-centric metabolomics-based approaches use sets of LC-MS/MS samples, analyze them with molecular networking,^18^ and correlate the bioactivity of the samples to the intensity of metabolite features within the samples.^19–21^ A key feature to identify the active components is hereby the correlation between the intensity of the metabolites and their activity signal. However, analyzing complex samples, or large fraction sizes, typically results in substantial signal overlap, which limits the unambiguous identification of the active component.

Here, we report on the development of a compound-resolved bioactivity-based LC-MS/MS approach radically shrinking the fraction size to achieve high-resolution bioactivity data, at the same frequency as the scanning of MS duty cycle. This enables more precise correlation of MS features to bioactivity signals and reduces ambiguity when associating with analytes. Our technology makes use of four components: 1. A custom high-speed fractionation device that is coupled to a commercial LC-MS/MS system. 2. Paper-based microfluidic devices (μPAD) that serve as microfractionation wells. 3. Bioreporter strains which can be deployed onto the μPAD and which produce a luminescence signal. And 4. custom software to read out the luminescent signal and correlate it to MS features. A conceptual overview of the system and approach is shown in Figure 1. After the development of the instrumentation, bioreporter strains and software, we validated the workflow with a panel of antibiotic standards. To showcase the practicality of the method we further screened bacterial crude extracts for antibiotics, during which we identified multiple metabolites with antimicrobial activities.

**Figure 1:**
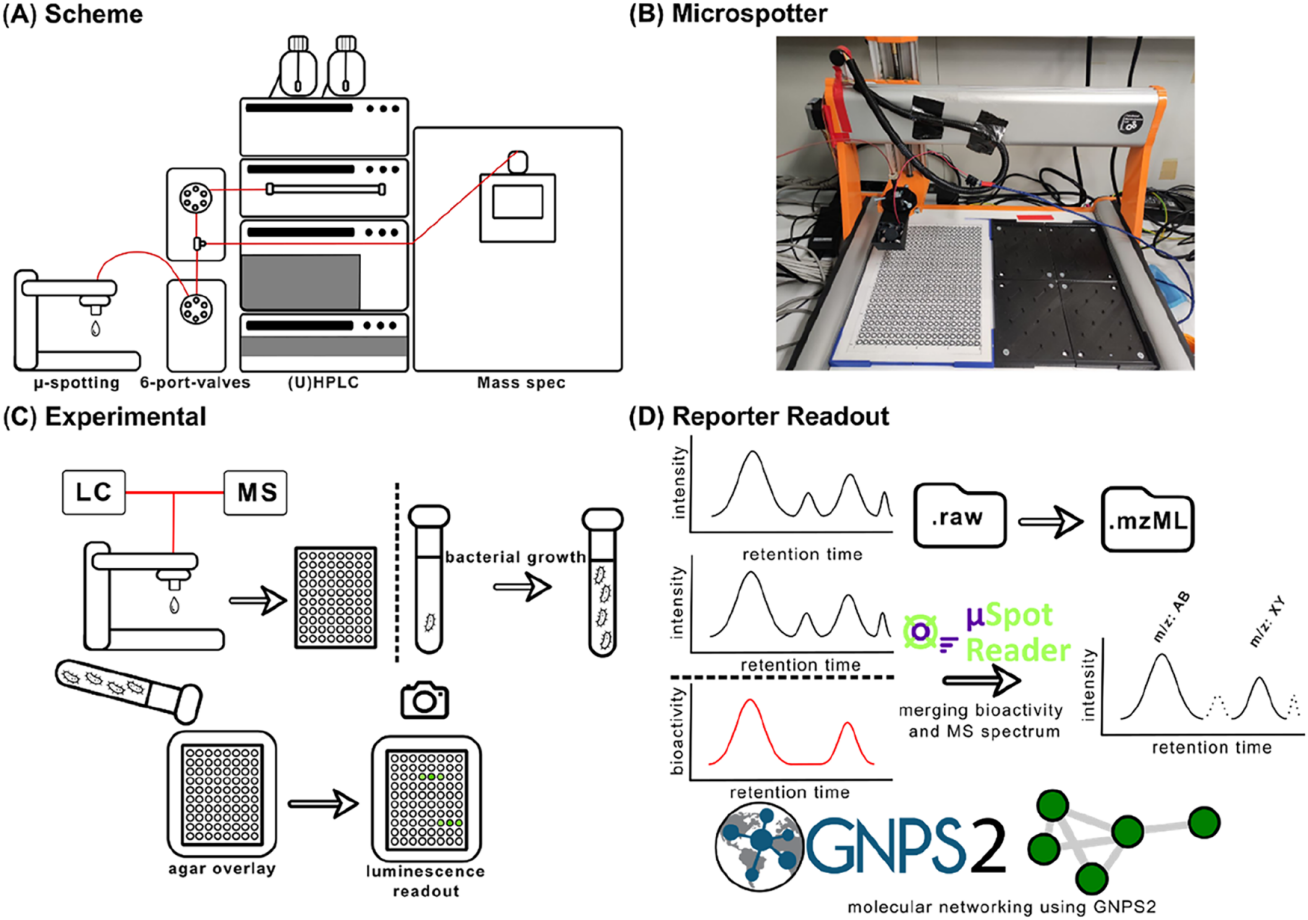
(A) Instrumental setup used for the experiments. Relevant capillaries are shown in red. Extracts are separated on an HPLC system and eluted into a double 6-port-valve setup. Between both valves is a T-piece. Here, the flow gets diverted between the MS and the Microspotter. (B) Image of the Microspotter. For detailed operation of the instrument, see Video S1 or https://www.youtube.com/watch?v=PTC9epQNzow. (C) Wet lab workflow for the use of the Microspotter. The extract is separated by LC. Subsequently, MS spectra are recorded and the eluate is spotted on a μPAD. In parallel, the bacterial bioreporters are grown. A spotted and dried μPAD is overlaid with agar containing the bioreporter strain. Luminescence readout can be performed after 180 min. (D) Dry lab workflow. The MS output-file is converted to .mzML format and fed into the MicrospotReader app, which is able to create a chromatogram based on the bioactivity data from the luminescence readout. Furthermore, it merges this chromatogram with the MS output file to filter only relevant *m*/*z*-values for the bioactivity. The app output file can be used for molecular networking using GNPS2.

## Experimental Section

### Materials

Chromatographic separation was performed on a Kinetex EVO LC column by Phenomenex (Torrance, California, USA). Specifications of the column were as follows: 150 mm × 4.6 mm (l. × i.d.), equipped with fully porous 2.6 μm particles with a pore size of 100 Å. Water was of Optima LC/MS-grade quality and was supplied by Fisher Chemical (Fisher Scientific, Hampton, NH, USA). Acetonitrile and formic acid was LC-MS grade and was supplied by Thermo Fisher Scientific (Bremen, Germany). All used antibiotic standards can be found in Table S1. The polylactic acid (PLA) filament for the 3D-printer was supplied by Labists (Shenzhen, China). Used oligonucleotides can be found in Table S2.

### Instrumentation

The microfluidic paper analytical device (μPAD) was printed using a wax printer (Xerox Colorqube 8580) from Xerox (Norwalk, CT, USA) or a thermal transfer printer (HPRT MT800) from Xiamen Hanin Electronic Technology Co., Ltd. (Xiamen, China). Hydrophobic “caps” were printed using the toner printer TASKalfa 352ci from Kyocera (Kyoto, Japan). The Microspotter is a three-axis robot, a modified milling machine from Stepcraft GmbH & Co. KG (Meden, Germany). 3D printing was performed using an i3 mega printer from Anycubic (Shenzhen, China) using polylactic acid (PLA) filaments. As HPLC, an Agilent 1260 Infinity II system from Agilent Technologies (Waldbronn, Germany) with a quaternary pump, an autosampler, and a UV/Vis detector was used. MS measurements were performed on a Q Exactive orbitrap system from Thermo Fisher Scientific (Bremen, Germany). Two 6-port valves were used in the instrumental setup, both Rheodyne valves from Idex (Northbrook, IL, USA). The imaging system for luminescence pictures was a ChemiDoc MP by Bio-Rad (Hercules, CA, USA).

### Fabrication of μPADs

Microfluidic paper-based analytical devices (μPADs) have gained a certain popularity since their introduction in 2007.^22^ They are inexpensive, easy to manufacture, and offer a versatile platform for biosensing, similar to the “lab-on-a-chip” approach.^23^ While colorimetric detection is commonly used,^24^ μPADs also support electrochemical^25^ and fluorescent methods,^26,27^ broadening their range of applications. A key advantage is their customizability: μPADs can be tailored to specific analytical needs. The operating mode is straightforward: a desired design is printed onto a commercially available sheet of paper using a solid ink printer. This forms a thin layer of a waxy resin-based polymer on top of the paper that is sensitive towards heat. When placing the printed paper into an oven at 110 °C for 1 min, the “wax” melts and migrates into the paper, forming a hydrophobic barrier. Here, 500 small circles were printed onto the paper, forming space for up to 500 fractions from the eluate in one LC-run. For high resolution fractionation with a spotting frequency of 1 Hz, a total of 8 min and 20 s of the LC run can be covered. The number of spots can individually be changed, but should be in accordance with the LC method. A sampling frequency of 1 Hz was chosen, as we expected the width of elution peaks to be wider than 1 s. When diverting the dead time (1 min) into the waste, almost the whole gradient time of 10 min can be covered by 500 spots.

To prevent compounds from migrating through the paper, “caps” were printed on the back of the μPAD after curating it at 110 °C. Those caps were not heated and stayed on the lower surface of the μPAD to prevent the spotted compounds from bleeding through the paper and diffusing into the agar under the PAD. Several different setups were tested, with this “capped” μPAD resulting in the most promising approach. A laser printer was used for the printing of hydrophobic caps. To be able to replace outdated solid-ink printers, a comparable workflow was also performed with a thermal transfer printer (see SI).

### Fabrication of High-Speed Microspotting Device (Microspotter)

We built the Microspotter on the basis of a commercial CNC milling machine (STEPCRAFT D.600 CNC System). For our purpose, the milling head was removed and replaced by a tailor-made 3D-printed head, with an adjustable PEEK capillary holder. The spotter head of the Microspotter precisely positions the outlet of the capillary through its three-axis motion from one spot to the next spot. For precise positioning of the eluent on the μPAD, we engineered a nozzle consisting of a PEEK capillary (i.d. 0.13 mm) within a 3D-printed spotter head. The spotter head consists of four parts (see Figures S2 and S3 and CAD files in data sharing section). Reliable hardware synchronization is essential for reproducible results and to avoid delay errors while spotting. To synchronize the MS with the Microspotter, a C++ embedded program was written to control an Esp32 microcontroller, which provided the contact closure for the spotter start. The code is publicly available and can be found in the data sharing section.

### LC-MS/MS and Microspotter Instrumental Setup

For the LC system, mobile phase A consisted of water +0.1% formic acid (V/V) and mobile phase B was acetonitrile +0.1% formic acid (V/V). Runtime was 15 min with the gradient as follows: 0.00 min: 5% B, 10.00 min: 95% B, 12.00 min: 99% B, 13.00 min: 99% B, 13.01 min: 5% B, flow rate 1 mL/min. Mass spectrometer conditions: full MS, scan range: *m*/*z* 150 to 2,000, resolution: 35,000, positive ion mode. Sheath gas: 35 psi, aux gas: 5 psi, sweep gas: off, spray voltage: 3.5 kV, capillary temperature: 250 °C, aux gas heater temperature: 250 °C.

Postcolumn, an automatic, time-programmable 6-port valve was installed. Here, the eluate can be directed either to the waste or to the MS and the Microspotter. In the latter line, a T-piece diverts the flow, 10% are infused in the MS, 90% are diverted to a second 6-port valve. This valve can be switched between waste and the Microspotter. When the first valve is in analysis-mode, the second valve can be switched to waste after spotting one μPAD. This ensures further infusion to the MS for the monitoring of, e.g., extremely lipophilic compounds that elute (under the chosen circumstances) only in very high percentage of organic solvent, usually at the end of a standard LC gradient. The arm of the spotter can be programmed for the *x*-, *y*- and *z*-axis and can precisely drive and stop over the printed spots of the μPAD. To precisely control the head of the three-axis-robot, HPLC outlet, and microspotting, the Microspotter was controlled using the CNC Drive software. Its real-time 3D toolpath viewer, friendly user-interface, and flexible machine control (programmable and manual) made it ideal for troubleshooting, testing, and ultimately programming a Microspotter motion profile. The motion profile is driven by G-code and coordinate-based commands to drive the robot’s movement. To streamline the coding process across similar motion profiles (e.g.,: same pattern but different starting coordinates or speed), a template was created using an R script.

### Bioreporter Design

Based on a sporulation-deficient mutant of the Gram-positive model organism *Bacillus subtilis* and established antibiotic stress-sensing promoters of the genes *fabHB, yorB, ypuA, liaI* or *bmrC*,^28–32^ we constructed five bioreporter strains that signal promoter activity using the bacterial luciferase system as a sensitive readout (Figure 2A).

**Figure 2:**
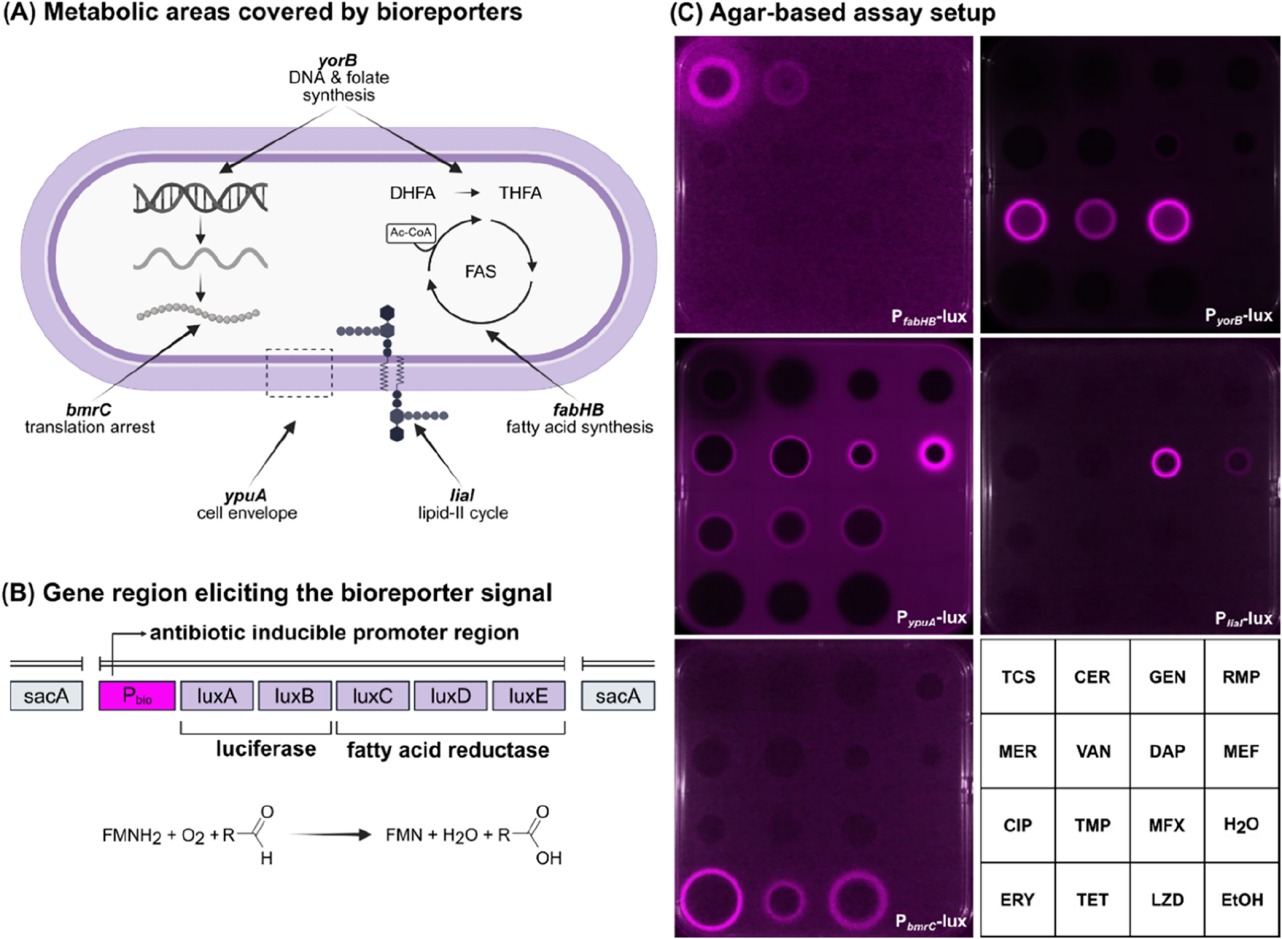
Biosynthetic pathway coverage, construction and validation of the bioreporter panel. (A) Schematic overview of the key biosynthetic processes covered by the bioreporter panel. Each bioreporter enables rapid detection of antimicrobial compounds interfering with the depicted major biosynthetic pathways: *yorB* (DNA and folate synthesis), *bmrC* (translation arrest), *liaI* (lipid II cycle), *ypuA* (cell envelope integrity), *fabHB* (fatty acid synthesis). DHFA, dihydrofolic acid; THFA, tetrahydrofolic acid; Ac-CoA, acetyl coenzyme A; FAS, fatty acid synthesis. (B) Schematic illustration of the gene region eliciting the bioreporter signal, integrated into the *sacA* locus of the sporulation-deficient *B. subtilis* 1S34 strain. For each bioreporter, the respective antibiotic-inducible promoter region (P_*fabHB*_, P_*yorB*_, P_*ypuA*_, P_*liaI*_, P_*bmrC*_) was cloned upstream of the *P. luminescens* *luxABCDE*-operon, consisting of a heterodimeric luciferase protein (LuxAB) and a fatty acid reductase complex (LuxCDE).^37^ Enzymatic conversion of long-chain fatty aldehydes in the presence of molecular oxygen and reduced flavin mononucleotide (FMNH_2_) promotes an autonomous production of light with a wavelength of 490 nm.^31,37^ (C) Representative images of the agar-based bioreporter assay exemplifying inductions of the bioreporter panel (P_*fabHB*_-lux, P_*yorB*_-lux, P_*ypuA*_-lux, P_*liaI*_-lux, P_*bmrC*_-lux) following exposure to 14 exemplary reference compounds with diverse mechanisms of action; for results on additional 36 agents, see Figure S5A. Antibiotics, denoted by their three-letter codes, were spotted onto the bioreporter lawn in a 4 × 4 grid at the following concentrations: triclosan (TCS), 2 μg; cerulenin (CER), 5 μg; gentamicin (GEN), 1 μg; rifampicin (RMP), 0.5 μg; meropenem (MER), 0.5 μg; vancomycin (VAN), 15 μg; daptomycin (DAP), 2 μg; mefloquine (MEF), 10 μg; ciprofloxacin (CIP), 1 μg; trimethoprim (TMP), 0.5 μg; moxifloxacin (MFX), 0.5 μg; erythromycin (ERY), 2 μg; tetracycline (TET), 20 μg; linezolid (LZD), 3 μg. Water and ethanol (5 μL each) served as solvent controls. Bioreporter induction was quantified by luminescence imaging at 180 min after antibiotic treatment, with luminescent halos indicating bioreporter activation.

The genes for the β-ketoacyl–acyl carrier protein synthase III *fabHB* were identified as a specific biomarker for fatty acid biosynthesis inhibition.^29,32–34^ While the primary function of YorB remains unknown, it is regulated by LexA and associated with the bacterial SOS response, serving as an indicator of DNA damage and folate synthesis interference, the latter via inhibition of nucleic acid synthesis.^28,32,35^ YpuA is implicated in general cell envelope stress responses and detects antibiotics disrupting the cell wall and membrane, whereas LiaI is more specific for undecaprenyl phosphate cycling inhibition (“lipid II cycle”).^28,29,31,32^ The subunit of a multidrug ABC transporter BmrC is natively expressed during late-exponential and stationary growth phase and serves as a biomarker for translation-arresting antibiotics.^29,36^ For each bioreporter, the corresponding antibiotic-inducible promoter (P_*fabHB*_, P_*yorB*_, P_*ypuA*_, P_*liaI*_, P_*bmrC*_) was cloned upstream of the *Photorhabdus luminescens* *luxABCDE*-operon and integrated into the nonessential *sacA* locus of the *B. subtilis* chromosome, enabling the stable detection of promotor induction. The *luxABCDE*-operon encodes a heterodimeric luciferase (LuxAB) and a fatty acid reductase complex (LuxCDE), enabling an autonomous production of luminescence through the oxidation of long-chain aliphatic aldehydes (Figure 2B). Details on the cloning procedure for the new bioreporter set are provided in the Supporting Methods section.

Autonomous light production by the stressed bacteria, i.e., independent of the addition of an external substrate, combined with the use of a sporulation-deficient *B. subtilis* background, avoiding the contamination of instrumentation by heat-resistant endospores, makes the new bioreporter set particularly suitable for the Microspotter application.

The bacterial luciferase-based bioreporters offer continuous self-sustained signal output, enabling a dynamic, time-resolved analysis, eliminating the necessity of external substrate addition as required for conventional firefly luciferase or β-galactosidase reporters. This novel bioreporter panel supports convenient whole-cell screening in both liquid and solid media.

### Bioreporter Assay Setup

Bioreporters are suitable in an agar-based assay format as exemplified in Figure 2C (for the full data set, see Figure S5A) and in a liquid assay setup (Figure S5B). Bioreporter cultivation was generally performed in lysogeny broth (LB; 1% NaCl, 1% tryptone, 0.5% yeast extract, pH 7.25) supplemented with 5 μg/mL chloramphenicol at 37 °C and shaking (190 rpm). For the agar-based assay, a liquid preculture was initiated for each bioreporter from a glycerol stock and incubated for 18 h, followed by inoculation of a main culture to an initial OD_600_ of 0.05, which was subsequently grown to an OD_600_ of approximately 1.0. From the main culture, soft agar (0.75% agar, without chloramphenicol) was inoculated to achieve a final cell count of 3 × 10^6^ colony-forming units (CFU)/mL. LB soft agar was used for P_*fabHB*_-lux, P_*yorB*_-lux, P_*ypuA*_-lux, P_*liaI*_-lux, while BMM soft agar^38^ was used for P_*bmrC*_-lux. The bioreporter soft agar, containing an individual bioreporter strain was poured over the μPAD, which had been previously placed into a square 120 mm × 120 mm Petri dish (Sarstedt) covered with a thin bottom layer of soft agar without bacterial cells. After a solidification period of 20 min, the bioreporter plates were incubated for 180 min at 37 °C. Luminescence was recorded using a ChemiDoc MP system (Bio-Rad) with an exposure time of 600 s in Chemiluminescent Blot 647SP mode, capturing light emission produced at a wavelength of 490 nm. Postimaging, the bioreporter plates were further incubated for 18 h to identify antimicrobial effects (inhibition zones). Incubation was performed at 30 °C for the strains P_*fabHB*_-lux, P_*yorB*_-lux, P_*ypuA*_-lux, P_*liaI*_-lux, while P_*bmrC*_-lux was incubated at 37 °C. For bioreporter validation by reference compounds, the bioreporter soft agar was poured into an empty Petri dish and pure compounds (≤3 μL, dissolved in DMSO) were directly spotted on the solidified soft agar and left to dry for 5 min before incubation. For the procedure of the liquid assay format see Supporting Methods.

### *Saccharopolyspora erythraea* Cultivation and Extraction

*Saccharopolyspora erythraea* was cultivated on ISP2 agar plates (0.4% d-glucose, 1% malt extract, 0.4% yeast extract, 2% agar, pH 7.3) for 7 days at 28 °C. Biomass was harvested using a cell scraper (Sarstedt) and transferred into a mixture of water and ethyl acetate (50/50, V/V). Extraction was performed in an overhead shaker for 18 h, followed by phase separation via centrifugation (4000*g*, 10 min, 4 °C). The organic phase was lyophilized and reconstituted in 80% (V/V) methanol.

### Data Analysis via the MicrospotReader and GNPS2

We developed a Python-based web application called MicrospotReader for data processing and integration of bioluminescence readouts and MS/MS data. The software is built as a streamlit web application and uses the NumPy,^39^ pandas,^40,41^ SciPy,^42^ scikit-image,^43^ Numba^44^ and pyOpenMS^45,46^ packages. The data processing is split into three steps: (I) Image analysis (II) activity data preparation and (III) LC-MS feature detection and correlation with activity data. We provide jupyter notebooks for each step of the process as well as a browser-based web app built using the streamlit framework.

(I) During image analysis activity values are derived automatically from the luminescence readout for each fraction by taking the mean pixel intensity of a given spot. The sizes of luminescent halos can be quantified if present in the image. In addition to overall luminescence intensity, the sizes of halos can be used to provide a semiquantitative estimate of bioactivity. Bioactivity values for all fractions are normalized by the median bioactivity of the image, ensuring comparability among samples.
(II) From the bioactivity data an activity chromatogram is constructed by assigning each fraction a retention time based on experimental parameters. The chromatogram is smoothed using a one-dimensional Gaussian kernel with a sigma-value of 1.
(III) We detected mass spectrometry features in the .mzML files using an untargeted metabolomics workflow provided by the pyOpenMS library.^45,46^ Bioactivity peaks are detected within the bioactivity chromatogram and correlated with mass features based on retention time and peak-shape correlation using a Pearson correlation. The retention time overlap is determined by considering a set bias and time window. Correlated features are ranked based on the Pearson correlation coefficient and correlations above 0.8 were kept. A visual representation of the processing workflow is shown in Figure S1.

Molecular networking was performed in GNPS2 (gnps2.org), using the classical networking workflow. Parameters were as follows: Precursor and fragment ion tolerance: 0.002, min cluster size: 2, min cosine (library and network): 0.7, min matched peaks (library and network): 6.

## Results and Discussion

### Microspotter Workflow

The concept of the presented compound-resolved bioactivity-based metabolomics workflow is based on parallelizing LC-MS/MS and biological activity data streams. The combination of both MS/MS and bioactivity signals over a chromatographic time dimension (Figure 1D) is achieved through high-frequency microfractionation and simultaneous MS and MS/MS analysis of the split LC flow (Figure 1A). To achieve fractionation frequencies in the same range as the MS/MS duty cycle time (∼1 Hz), we built a custom Microspotter device based on a commercial CNC milling instrument (Stepcraft, see experimental section, Figures 1B and S2 for details). The stepping motors of the Microspotter allow for a fast and precise switching between the spots without losing eluate despite constant flow (without an additional valve at the capillary outlet). Due to the spotting speed droplets are formed on the tip of the capillary at the spotter head and are deposited onto the μPAD before droplet separation takes place. Transfer of the hanging drops is performed by a lowering of the spotting head into *z*-axis direction, so that the drop, but not the capillary comes into spatial contact with the μPAD (for details, see Video S1 or https://www.youtube.com/watch?v=PTC9epQNzow). The hydrophobic barriers of the μPAD contain the fractions of LC, where the mobile phase can rapidly evaporate, before they can be further tested for their bioactivity. This is crucial, as organic solvents can lead to false positive hits by interfering with bacterial cell viability. To assess possible spot-to-spot carryover and diffusion, we performed further experiments using flow injections (see Figure S8 and subchapter “spot-to-spot carryover in the SI), which indicated that division effects of the Microspotter and related spot-to-spot carry over, were in a similar range as for the ESI-MS part of our setup. In the here described search for antibacterial activity, we overlaid the dry μPADs with bioreporter-containing agar for rapid and sensitive detection of cellular stress that is indicated through the expression of luciferase and the emission of light. The individual steps of the procedure (Figure 1C) may be split into two separate workflows and can be performed in parallel.

The use of bioreporters has several benefits compared to the nonengineered wild-type strains commonly used in bioactivity assays. Reading out a rapid physiological reaction by sensitive luminescence detection yields reliable data already after 180 min, while standard bioactivity assays are generally assessed after 18–24 h. The stress responses signaled by the bioreporters are already triggered at sublethal antibiotic concentrations, resulting in higher sensitivity than the classical growth inhibition readout (see Figure S4). Consequently, bioreporters are superior to classical test strains in detecting low abundant or weakly active agents. Another advantage results from our use of not a single bioreporter, but a defined set of closely related strains that report on different cellular modes of action of antibacterial agents. An individual light-emitting strain among the set informs about the target area of the antibacterial compound, which can assist in the dereplication of known compounds and guide potential downstream assays to identify the molecular target(s) of novel compounds.

After incubating the μPADs with the bioreporter strains, luminescence images are taken, and together with the corresponding LC-MS/MS files, serve as input files for the subsequent computational data analysis. We developed a web application (microspotreader.gnps2.org) to first perform a densitometry analysis of the bioluminescence image, which is then transformed into a bioactivity chromatogram that can be correlated against all extracted ion chromatograms (XICs) from the LC-MS/MS data to identify overlapping features (Figure 1D). The app’s output data can then be used to perform molecular networking in GNPS2. In this way, it can be seen if spectra of compounds that are already known to the database were acquired. Furthermore, compounds with spectral similarity cluster in a network and can be interpreted as derivatives.

### Bioreporter Induction Specificity

To validate the specificity of the bioreporter panel, we used a collection of 50 reference antibiotics with well-characterized and diverse mechanisms of action (Table S1). Figure 2C exemplifies a representative subset of reference agents in the agar-based setup, and the full data set is shown in Figure S5A. In the agar-based assay, bioreporter induction was quantified by luminescence imaging after 180 min of antibiotic exposure. Within this time frame, the bioreporter signals related specifically and robustly to the agents with the corresponding mechanisms of action, while prolonged incubation occasionally resulted in nonspecific responses. In the agar-based assay, luminescent halos indicating bioreporter activation appeared at the margin of zones of growth inhibition, i.e., at compound concentrations in the diffusion gradient that triggered the bacterial stress responses but still allowed for bacterial metabolism to generate the luciferase signal. The luminescent halos were subsequently superimposed as a pink colorization onto an epi-luminescence image capturing zones of growth inhibition. By relying exclusively on bioluminescence instead of fluorescence, the current system also avoids interference from autofluorescent natural products.

The specificity of the bioreporters was validated in previous studies^28,29^ using over 100 reference antibiotics with defined modes of action, ensuring that only promoters responding selectively to their intended target areas were used. In the current work, the same validated promoters were used to drive bacterial luciferase expression, and their specificity was reconfirmed with reference antibiotics, confirming the absence of leakiness or crosstalk.

The bioreporter P_*fabHB*_-lux was selectively induced by the fatty acid synthesis inhibitors triclosan^47^ and cerulenin^48,49^ (Figures 2C and S5A). The activity of DNA gyrase inhibitors, nucleotide synthesis inhibitors, and DNA intercalating agents were detected by P_*yorB*_-lux (Figure S5A). In Figure 2C, both DNA gyrase inhibitors ciprofloxacin and moxifloxacin,^50–53^ as well as the nucleotide synthesis inhibitor trimethoprim^54,55^ show strong inductions. Additionally, P_*yorB*_-lux responded to nitrofurantoin, a pleiotropic antibiotic known to also interfere with DNA.^56,57^ The general cell envelope stress bioreporter P_*ypuA*_-lux reacted to compounds interfering with enzymes and precursors in peptidoglycan synthesis, cell membrane disruptors and certain ionophores (Figure S5A). The phospholipid-binding membrane disruptor mefloquine^58^ elicited the strongest signal, followed by the lipid II cycle inhibitors vancomycin^59^ and daptomycin,^60,61^ and the peptidoglycan transpeptidase inhibitor meropenem.^62^ Notably, P_*ypuA*_-lux was also induced, albeit weakly, by DNA-active agents as a secondary mechanism of action, consistent with previous observations.^28^ This response may result from the activity of the SOS-induced cell division inhibitor YneA, which binds peptidoglycan and interacts with late divisome proteins, thereby inhibiting septal cell wall synthesis and cell division following DNA damage.^63^ In contrast, the bioreporter P_*liaI*_-lux was induced on agar only by the lipid II cycle inhibitor daptomycin (DAP) as well as mefloquine (MEF) (Figures 2C and S5A). The P_*bmrC*_-lux bioreporter was activated by antibiotics that cause translational arrest through diverse mechanisms (Figure S5A). In Figure 2C, bioreporter induction by the exit channel blocker erythromycin,^64^ tRNA binding inhibitor tetracycline,^65^ and translation initiation inhibitor linezolid^66^ is exemplified. Notably, the activation of P_*bmrC*_-lux was specific to translational arrest, whereas agents causing miscoding (e.g., gentamicin), general protein-stress (e.g., acyldepsipeptides, ADEPs), abortive translation (e.g., puromycin) or tRNA synthetase inhibitors (e.g., mupirocin) did not induce P_*bmrC*_-lux (Figure S5A), corroborating previous findings.^28,29^ The bioreporter panel was further assessed in a second, independent whole-cell screening assay in liquid culture applying the same set of reference antibiotics (see Figure S5B and Supporting Methods). In summary, each bioreporter strain demonstrated a selective activation profile in accordance with the established modes of action of the reference antibiotics.

### Validation of the Microspotter-LC-MS/MS System

A primary investigation of the bioactive capabilities of the sample of interest is an important step to help reduce the downstream workload. For this purpose, we directly tested crude extracts on the entire bioreporter panel, by manually spotting 10–20 μL of the sample onto the μPAD, repeated once for each bioreporter strain (Figure S4). Generally, one of the bioreporters gave a response, rarely two due to overlapping modes of action. We recommend performing this as a first step in assessing unknown extracts, as it helps to efficiently pinpoint the bioactivities of the sample of interest.

Next, we validated the Microspotter setup using a mixture of three commercially available antibiotic compounds and the P_*yorB*_-lux bioreporter strain (DNA synthesis stress). The mixture containing ciprofloxacin (500 ng on column (o.c.)), moxifloxacin (250 ng o.c.) and trimethoprim (250 ng o.c.) was spotted applying the HPLC gradient described above. The data analysis pipeline is displayed in Figure 3, beginning with the luminescence image generated by incubating the μPAD for 3 h with the respective bioreporter strain (Figure 3A). From the 500 spots on the μPAD, only the first 200 are shown here. While the first minute (which roughly translates to the dead time of the system) was not spotted, the shown 200 spots represent the elution time from 1.00 to 4.33 min. Spotting was performed in a serpentine way to not lose resolution while changing to another row.

**Figure 3:**
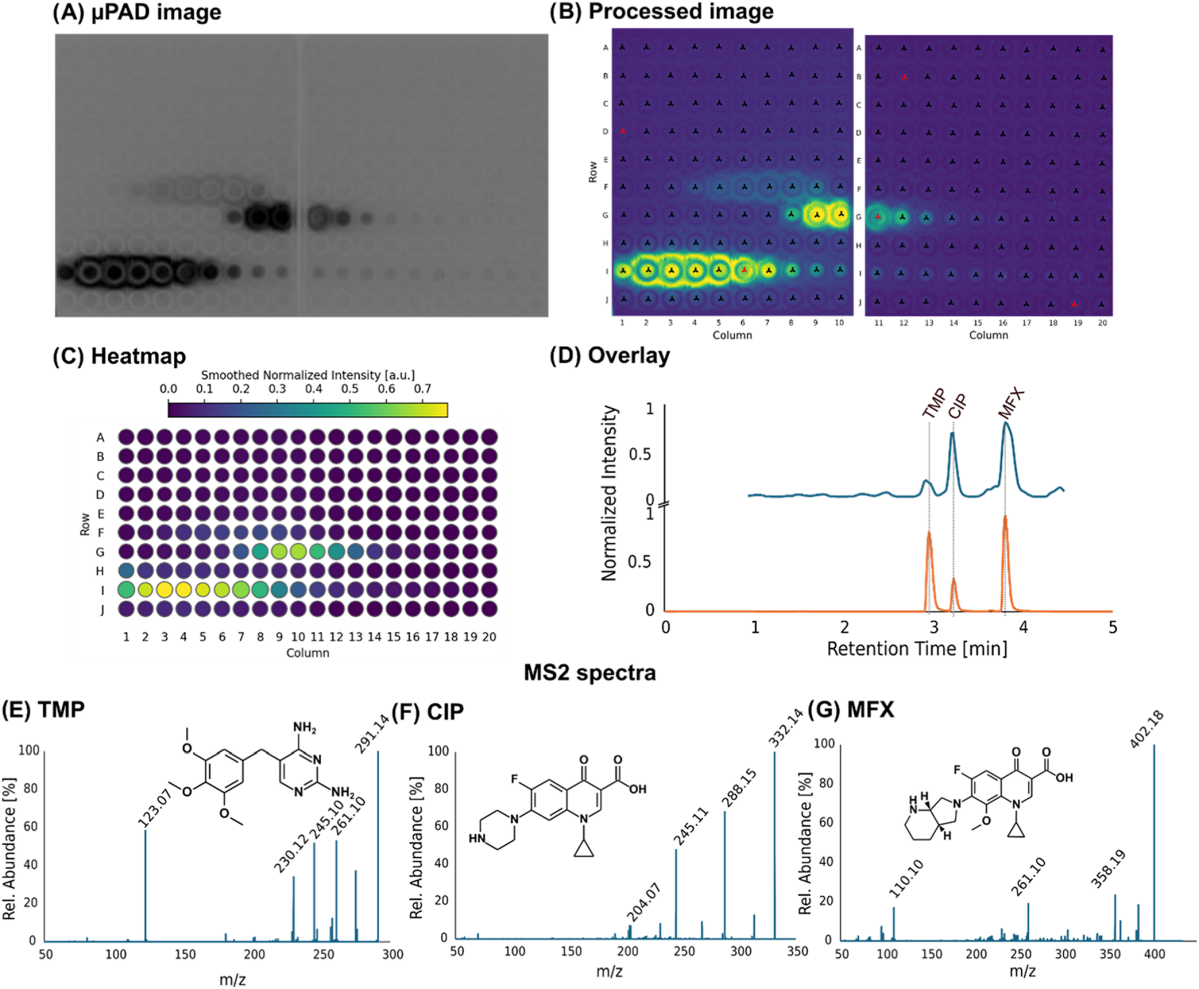
Workflow of the densitometric analysis of a spotted μPAD and its translation into a bioactivity chromatogram. (A) An image of the μPAD is generated, where luminescence can be displayed (here, colors are inverted to increase contrast). (B) The image is recognized by the app, spots are assigned to coordinates. The images will subsequently be transformed into a heatmap (C). By correlating every spot to a distinct retention time, a bioactivity chromatogram (D, blue trace) can be obtained. A comparison to the total ion current (TIC) as obtained with the MS (orange trace) can be performed by overlaying both chromatograms and by comparing the peak shape at a distinct retention time (D) (TMP, trimethoprim; CIP, ciprofloxacin; MFX, moxifloxacin). (E–G) shows the MS2 spectra (positive mode) of trimethoprim (E), ciprofloxacin (F) and moxifloxacin (G); collision energy for all: 35 eV.

Figure 3A clearly shows the luminescence traces that are baseline separated from each other. Figure 3B shows the recognition of the luminescence by performing a densitometric analysis by the use of the MicrospotReader web application (see Chapter 2.9 and Figure S1). As the spotted μPADs were incubated in squares of 10 × 10 spots, two images were taken separately to acquire the images shown in (A) and (B). In a next step, the app merges the information gained from the images to provide a heatmap of detected bioactivity, as the sum of luminescence signal and halo diameter (Figure 3C). By aligning the information shown in the heatmap with the retention time for each spot, the app can provide a bioactivity chromatogram (Figure 3D, blue trace). Using this noncomplex sample, a simple overlay of the bioactivity chromatogram with the total ion current (TIC, orange trace) leads to the detection of the bioactive masses. While the manual inspection is fairly easy in noncomplex samples, it can be hard to perform in complex samples. For this reason, the app uses further information, like peak shape, for the identification of the bioactive peak in complex samples with a multitude of coeluting features. The recording of MS2 spectra gives further valuable information on the detected bioactive compounds and facilitates the structure elucidation when a previously unknown compound is shown to be bioactive. In Figure 3E–G, the recorded MS2 spectra of the three bioactive compounds are shown.

### Assessment of Limits of Detection of the Bioactivity Readout

The strength of response by the bioreporter is dependent on the activity (i.e., antibacterial potency and specific mode of action) of the compound, as well as its concentration in the eluted fraction. Compounds with low bioactivity may yield a high signal if present in high amounts; contrarily compounds with high bioactivity may give rise to low signal if not present at sufficient concentration in the sample.

Here, substances with different modes of action were used, and a 2-fold dilution series was performed and manually spotted using a pipet. To simulate the spotting with the Microspotter—and by this the higher volume (roughly 15 μL per spot) and more homogeneous distribution of the substance over the surface of the spot—first 10 μL of a mixture of water/methanol (50/50, V/V) was manually spotted on the μPAD. Thereafter, 5 μL of the antibiotic were pipetted into the water/methanol mixture on the μPAD. Subsequently, the μPADs were left drying and then incubated with the bacteria. For each used bioreporter strain, one antibiotic was chosen, from which the lower limit of detection (LOD) values for luminescence readout, as well as inhibition zone, were determined from the same sheet. In Figure S4, it can clearly be seen that the LOD of the luminescence signal of the bioreporter-strains is lower than the LOD of a classical inhibition zone test. The inhibition zone after 18 h is most probably also dependent on the thickness of the agar layer, as classical inhibition zone tests are performed by spotting the antibiotic on top of the agar, not the other way round as in this case. Nevertheless, this result indicates that the sensitivity of the bioreporter based method is higher. This is mechanistically plausible, as the inhibition zone requires full growth inhibition of the bacteria, while the bioreporter strain-based method produces signals when the bacteria are stressed, but can still grow. This lowers the lower detection threshold, in accordance with previous observations.^28^ The plot of the area under the curve (AUC) as obtained by densitometric analysis against concentration reaches a plateau at a distinct concentration. This shows the reached saturation and by this the upper detection limit. LODs were as follows: moxifloxacin (1 ng/spot), erythromycin (1 ng/spot), triclosan (2 ng/spot), vancomycin (59 ng/spot), and daptomycin (125 ng/spot) (see Figure S4).

### Identification of Erythromycin as a Bioactive Component in the Extract from *Saccharopolyspora erythraea*

Next, we evaluated the usability of the Microspotter workflow by screening crude extracts from *S. erythraea*, a natural producer of macrolide antibiotics from the erythromycin family. An agar plate of *S. erythraea* was extracted, and the extract was spotted and subsequently overlaid and incubated with P_*bmrC*_-lux (translational arrest). After luminescence readout, we could identify bioactivity as shown in Figure 4A.

**Figure 4:**
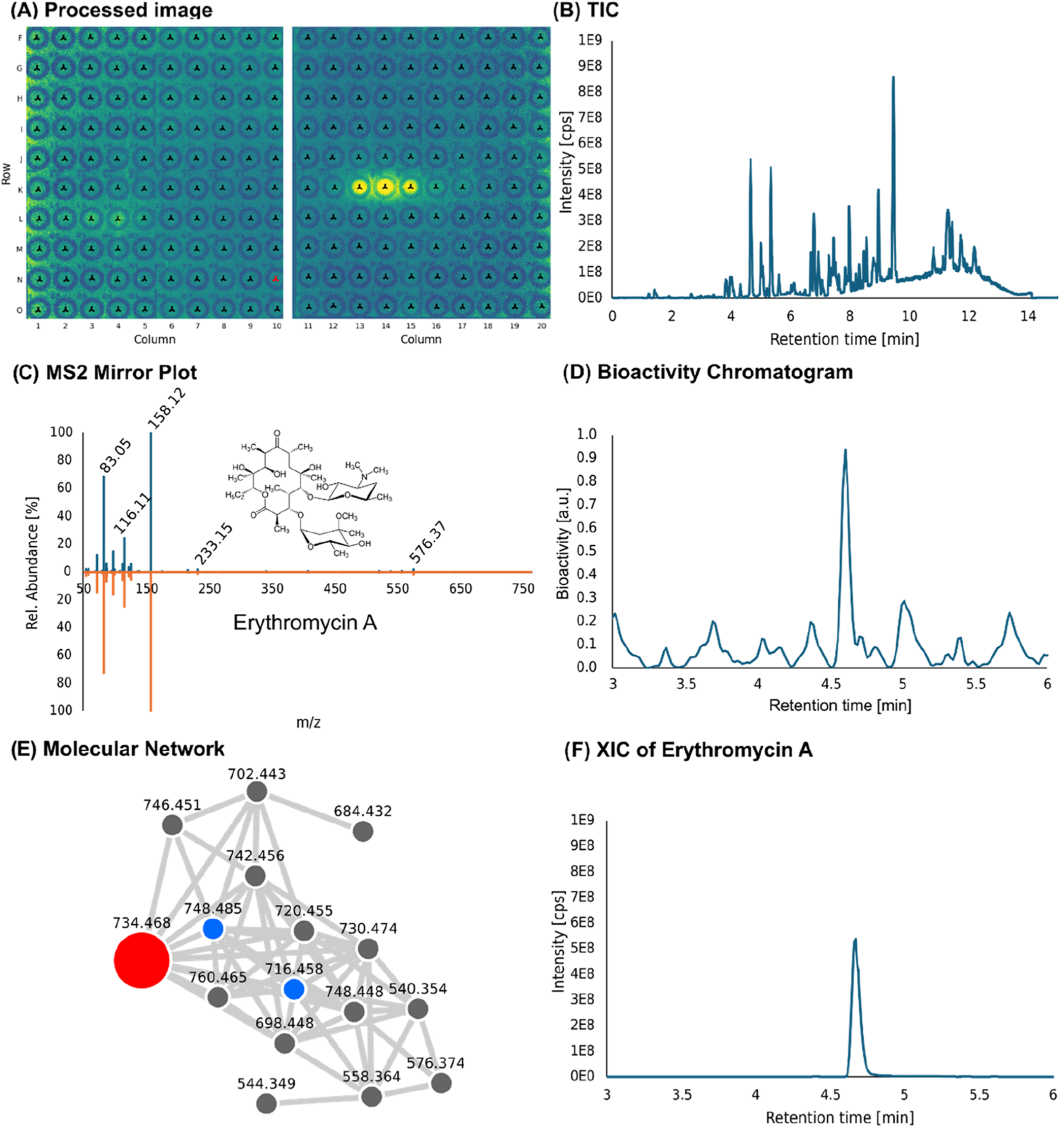
(A) Overview of the observed bioactivity on the μPAD spotted with bacterial extract from *S. erythraea*, shown are spots with a retention time of ∼2.6–6 min. (B) Total ion current (TIC) of the crude extract. (C) MS2 (mirror plot) of an authentic erythromycin A standard (upper part in blue) and the bioactive compound found in the crude extract (lower part, orange). (D) Bioactivity chromatogram obtained from (A). (E) Section of the molecular network generated with the data obtained with the extract from *S. erythraea*. The red highlighted node translates to a spectral match to erythromycin A, the blue nodes are spectral matches to an erythromycin derivative (anhydroerythromycin A) and erythromycin E. (F) extracted ion chromatogram (XIC) of erythromycin A.

A strong luminescence signal can clearly be seen in row K. In addition, spots L2–L4 show very light luminescence. Figure 4B shows the TIC of the extract, with (due to the extraction protocol) mostly mid- to nonpolar compounds. While D shows the bioactivity chromatogram, F shows the XIC of the correlating natural product, which corresponds to the mass of erythromycin A. Figure 4C shows a mirror plot of an MS2 spectrum of an authentic erythromycin A standard (upper part, blue) in comparison to the MS2 spectrum gained from the bioactive mass found in the extract, which further confirms the identity of erythromycin as level 2 ID.

In addition to erythromycin A, molecular networking (Figure 4E) showed that multiple compounds with spectral similarity were found in the extract as well. Here, the red node shows the bioactive compound that was annotated by library search on GNPS as erythromycin. Two further erythromycin derivatives were identified here via exact mass and MS/MS spectrum-library matching, namely anhydroerythromycin A and erythromycin E (see Figure S6).^67^ These results show that our bioactivity-based microfractionation approach, in combination with molecular networking is a powerful tool to quickly identify bioactive compounds, provide insights on their mode of action, and dereplicate their structure, or prioritize new chemical scaffolds for subsequent structure elucidation.

## Conclusion

We introduced a novel compound-resolved bioactivity-based metabolomics workflow, including customized open-source hard- and software for the accelerated discovery of bioactive metabolites. While the Microspotter was used in the discovery of antibiotic compounds in this study, it could also be exploited for other applications with other bioassays and formats. The μPAD can be adjusted to meet different assay needs, fit commercial fraction collectors, or be exchanged with 96 or 384 microwell plates for bioactivity assay in liquid state. Focusing on antibiotic activity, we constructed five nonsporulating bioreporter strains that respond to different cellular stress modes of action by autonomous light emission. We validated and tested the bioreporters and the Microspotter workflow with a panel of antibiotic standard compounds as a proof of principle. We could hereby unambiguously assign bioactivity to the masses and MS2 spectra of all standards. While further evaluating the performance and practicality of our approach with complex crude extracts from the actinomycete bacterium *S. erythraea*, we could rapidly identify the known macrolide antibiotic erythromycin A and derivatives.

In summary, our results demonstrate that LC-MS/MS coupled microfractionation on μPADs at high frequency and in combination with luminescent bioreporter strains is a powerful strategy for the search for novel bioactive compounds. We anticipate that its versatility and high-throughput capability will contribute to accelerate the discovery of bioactive metabolites from complex extracts.

## Supplementary Material





## Data Availability

All LC-MS/MS raw and processed files as well as luminescence images are available through the Zenodo (10.5281/zenodo.10800808) and MassIVE (MSV000094260) repositories. CAD files for the 3D printable parts of the Microspotter are available through the Zenodo repository (10.5281/zenodo.15796942) as well as on Thingiverse (thingiverse.com/thing:7077237/). GNPS2 molecular networking results of the crude extract of *S. erythraea* is available under: https://gnps2.org/status?task=0df811f274154242a7b349ac5e8d4b91. Code of the MicrospotReader (Knoblauch, S. (2025)). MicrospotReader WebApp (Version v0.1.1) can be found at: https://github.com/Functional-Metabolomics-Lab/MicrospotReader and a release has been deposited at Zenodo with the following DOI: 10.5281/zenodo.15494998. Executable files for local installations can be downloaded at: https://github.com/sknesiron/MicrospotReader/releases/tag/v0.1.1. Code for the Autostart (Fleischer, J. (2025) Microspotter-Synchronizing hard-/Software) can be found at https://github.com/Functional-Metabolomics-Lab/UCCNC_api_test and a release has been deposited at Zenodo with the following DOI: 10.5281/zenodo.15756837.

## Notes

Antibiotics used in this study, primers, microplate reader settings, figures for the app’s workflow, close-up picture of the tailor-made Microspotter head, images of the 3D-printable files, validation of the assay and determination of LODs, structure of erythromycin and derivatives, comparison of wax printed and thermal transfer printed μPADs, determination of possible spot-to-spot carryover and diffusion, methods for cloning of lux bioreporters and liquid based bioreporter assay, notes on comparison of wax printed and thermal transfer printed μPADs, Microspotter head fabrication and spot-to-spot carryover (PDF)
Microspotter working (Video S1) (MP4)
