# Supplementary material for: High-Frequency Microfluidic Fractionation for Compound-Resolved Bioactivity-Based Metabolomics
Source: Anal Chem. 2025 Oct 25;97(43):24093–104. doi: 10.1021/acs.analchem.5c04612 (PMC12590468; doi:10.1021/acs.analchem.5c04612)
Supplement: Supplementary file 1 [file ac5c04612_si_001.pdf]

Supplemental Information

High-frequency microfluidic fractionation for compound-resolved bioactivity-based metabolomics

Christian Geibel[1,2], Julian Schubert[1,2], Simon B. Knoblauch[2], Albert Hernandez[2], Leonardo Boldt[1,2], Dana C. Schneider[1,2,3], Stilianos Papadopoulos Lambidis[2,4], Giovanni Andrea Vitale[1,2], Jakub Fleischer[5], Manuela Haussmann[6], Harald Gross[2,3,6], Mingxun Wang[7], Heike Brötz-Oesterhelt[1,2,3,*], Daniel Petras[2,8,*]

1. Department of Microbial Bioactive Compounds, Interfaculty Institute of Microbiology and Infection Medicine (IMIT), University of Tübingen, 72076 Tübingen, Germany
2. Cluster of Excellence Controlling Microbes to Fight Infections (CMFI), University of Tübingen, 72076 Tübingen, Germany
3. German Center for Infection Research (DZIF), Partner Site Tübingen, 72076 Tübingen, Germany
4. Department of Microbiome Science, Max Planck Institute for Biology, 72076 Tübingen, Germany
5. University of Innsbruck, 6020 Innsbruck, Austria
6. Pharmaceutical Institute, Department of Pharmaceutical Biology, University of Tübingen, 72076 Tübingen, Germany
7. Department of Computer Science and Engineering, University of California Riverside, Riverside, California 92521, USA
8. Department of Biochemistry, University of California Riverside, Riverside, California 72521, USA

* Correspondence: heike.broetz-oesterhelt@uni-tuebingen.de or dpetras@ucr.edu

Table of Contents

Table S1: Antibiotics used in this study……………………………………………………...…S3
Table S2: Primers……………………………………………………………………………………S4
Table S3: Microplate reader settings………………………………………………………………S4
Figure S1: App workflow…………………………………………………………………………..S5
Figure S2: Microspotter head……………………………………………………………………..S5
Figure S3: Drawing of 3d-printable parts…………………………………………………………S6
Figure S4: Dilution series and LOD determination of tested antibiotics……………………..S7
Figure S5A: Agar-based specificity validation……………………………………………………S8
Figure S5B: Liquid-based specificity validation…………………………………………………S9
Figure S6: Erythromycin A and derivatives detected in *S. erythraea* crude extract……..S10
Figure S7: Comparison of wax printed and thermal transfer printed µPADs………..……S11
Figure S8: Determination of spot-to-spot carryover and diffusion………………..……..S12
Cloning of the Lux bioreporters…………………………………………………………………S13
Liquid-based bioreporter assay…………………………………………………………………S13
Comparison of wax-based and thermal transfer PAD…………………………………………S14
Microspotter head fabrication…………………………………………………………………...S15
Spot-to-spot carryover……………………………………………………………………………S15
Supplementary References………………………………………………………………………S16

Table S1: Antibiotics used in this study.

Antibiotic	Solvent	Supplier
**Fatty acid synthesis inhibitors**		
Cerulenin	DMSO	Cayman Chemicals
Triclosan	DMSO	LKT Laboratories
**DNA synthesis inhibitors**		
DNA gyrase binders		
Ciprofloxacin	H_2O	AppliChem
Moxifloxacin	H_2O	Fluka
Norfloxacin	DMSO	Fluka
Nalidixic acid	DMSO	Roth
Novobiocin	H_2O	AppliChem
Intercalators		
Phleomycin	H_2O	Invitrogen
Proflavine	H_2O	Sigma-Aldrich
Nucleotide synthesis inhibitors		
Azaserine	H_2O	Cayman Chemicals
Sulfamethoxazol	DMSO	Sigma-Aldrich
Trimethroprim	DMSO	Sigma-Aldrich
Pleiotropic effects		
Nitrofurantoin	DMSO	Sigma-Aldrich
**Cell envelope inhibitors**		
Peptidoglycan synthesis enzyme inhibitors		
Fosfomycin	H_2O	Sigma-Aldrich
Penicillin G	H_2O	Fluka
Methicillin	H_2O	Sigma-Aldrich
Ampicillin	H_2O	Roth
Cefadroxil	H_2O	Sigma-Aldrich
Cefotaxime	H_2O	Sigma-Aldrich
Cefuroxime	H_2O	Sigma-Aldrich
Meropenem	H_2O	Fluka
Peptidoglycan precursor binders		
Vancomycin	H_2O	AppliChem
Teicoplanin	H_2O	Aventis Pharma
Daptomycin	H_2O	Cayman Chemicals
Cell membrane disruptors		
Colistin	H_2O	Cayman Chemicals
Mefloquine	DMSO	Sigma-Aldrich
Ionophores		
Salinomycin	Ethanol	Cayman Chemicals
CCCP	DMSO	Sigma-Aldrich
Efflux pump inhibitors		
Reserpine	DMSO	Fluka

Antibiotic	Solvent	Supplier
**Protein synthesis inhibitors**		
Translation stallers		
Linezolid	DMSO	Acros Organics
Anhydrotetracycline	DMSO	Cayman Chemicals
Tetracycline	DMSO	Sigma-Aldrich
Doxycycline	H_2O	AppliChem
Chloramphenicol	Ethanol	Sigma-Aldrich
Clindamycin	H_2O	Cayman Chemicals
Lincomycin	H_2O	Alfa Aesar
Erythromycin	DMSO	AppliChem
Telithromycin	DMSO	Cayman Chemicals
Fusidic acid	H_2O	AppliChem
Hygromycin B	H_2O	Roth
Thiostrepton	DMSO	AppliChem
Miscoding inducers		
Gentamycin	H_2O	AppliChem
Kanamycin	H_2O	Roth
Tobramycin	H_2O	Acros Organics
Abortive translation inducer		
Puromycin	H_2O	Sigma-Aldrich
Protein stress inducer		
TMAD	H_2O	Sigma-Aldrich
tRNA synthetase inhibitors		
Mupirocin	H_2O	Sigma-Aldrich
Antibiotic	Solvent	Supplier
**RNA polymerase binders**		
Fidaxomicin	Methanol	Cayman Chemicals
Rifampicin	DMSO	AppliChem
**Diverse mechanism of action**		
Clp protease dysregulators		
Acyldepsipeptide 2	DMSO	EMC Microcollections
Acyldepsipeptide 7	DMSO	EMC Microcollections

Table S2: Primers.

PCR primer	Nucleotide sequence (5’-3’)
PyheI-forward	GATAAGCTGTCAAACATGAGAATTCTTCTTACTATTTTCACTTCCGTCAAACG
PyheI-reverse	TTTCATAGAGAGTCCTCCTGTCGACCTCATCAGCCGCCTTCTATTTTTTC
PfabHB-forward	GATAAGCTGTCAAACATGAGAATTCTCATAGATTCCTATCTACACTTCTC
PfabHB-reverse	TTTCATAGAGAGTCCTCCTGTCGACCACTCCTTATGGTCAGATTATAACAC
PliaI-forward	GATAAGCTGTCAAACATGAGAATTCCGGGTATCGGAATCTTGCTGTTTAC
PliaI-reverse	TTTCATAGAGAGTCCTCCTGTCGACTCCAAAAAAGACGGAGATCCCAAATAC
PyorB-forward	GATAAGCTGTCAAACATGAGAATTCCGGGATATATTGGGATAAAGATTCAG
PyorB-reverse	TTTCATAGAGAGTCCTCCTGTCGACTTTTGAAATTTTTGGTACTACTAAATTAT ATACC
PypuA-forward	GATAAGCTGTCAAACATGAGAATTCGCGGGCATCCGCCTTGGCTGACGAA
PypuA-reverse	TTTCATAGAGAGTCCTCCTGTCGACCAATTTCACAAGCAGCTGGATAGTGCTG CTTTGTG

Sequencing primer	Nucleotide sequence (5’-3’)
Insert-control-forward	TTCGTTTGTTGAACTAATGGGTGC
Insert-control-reverse	AAACCACACTCCTCAGAGATG
Insert-yheI-reverse	TCGACCTCATCAGCCGCCTTC
Insert-fabHB-reverse	TCCTGTCGACCACTCCTTATG
Insert-liaI-reverse	AAGACGGAGATCCCAAATAC
Insert-yorB-reverse	TGCACCAATACCTTGAACATC
Insert-ypuA-reverse	TCGACCAATTTCACAAGCAGC

Colony PCR primer	Nucleotide sequence (5’-3’)
SacA-Int-forward	CTGATTGGCATGGCGATTGC
SacA-Int-reverse	ACAGCTCCAGATCCTCTACG

Table S3: Microplate reader settings.

Parameter	Settings
Lid	Yes
Humidity cassette	No
Temperature control	On - wait for temperature
Temperature	37°C [min: 36°C, max: 38°C]
Measurement duration	Three hours
Interval time	Five minutes
Interval ▼	
Shaking	60 s, double orbital, amplitude 2.5 mm (108 rpm)
Wait	10 s
Luminescence	Integration time 1000 [ms]
Absorbance	Wavelength 600 nm, bandwidth 3.5, Flashes 10, settle time 100 [ms], multiple reads per well: XY-line 3x3, border 2400 [µm]
Wait	Wait for interval restart

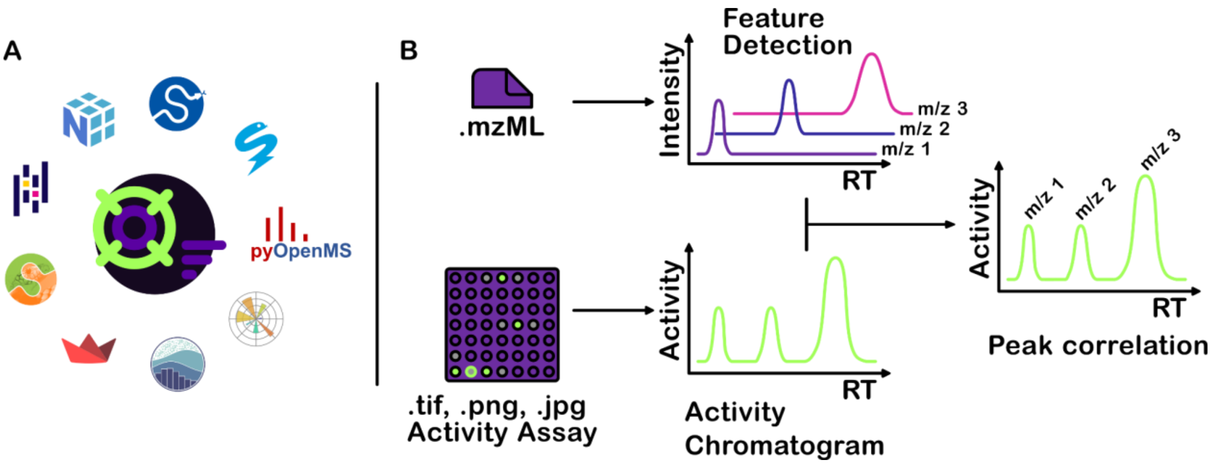

Figure S1: A) MicrospotReader leverages multiple optimized libraries for data processing (pandas, NumPy, SciPy, Numba, scikit-image, pyOpenMS), data visualization (Seaborn, matplotlib) and streamlit as a web app framework. B) Feature detection in LC-MS data (top), construction of an activity chromatogram from a bioactivity assay on LC-fractions (bottom) and correlation of activity peaks with LC-MS features (right).

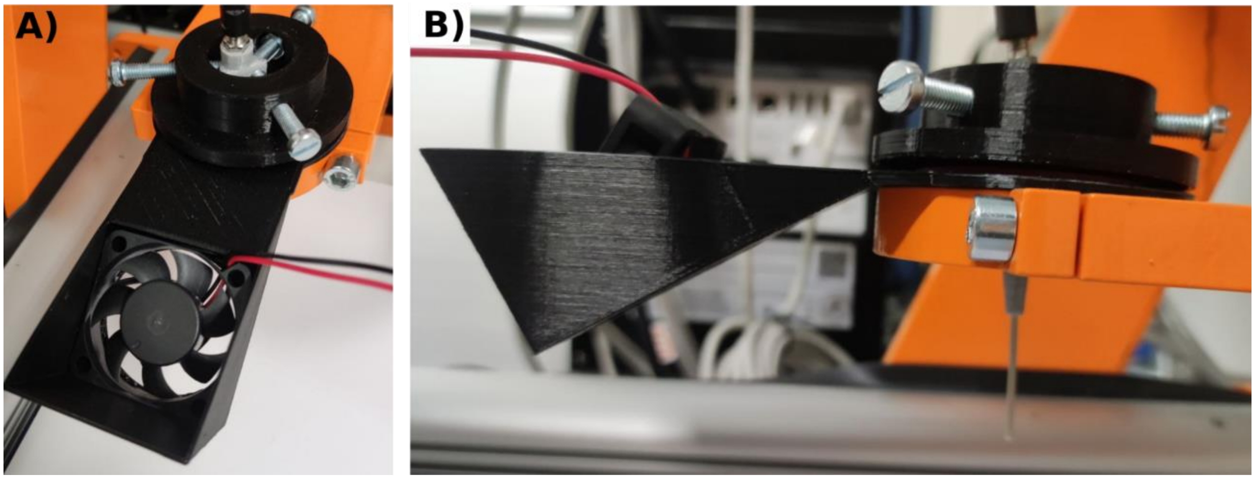

Figure S2: Tailor-made Microspotter head, A) top view with cooling fan (lower part) and adjustable capillary holder (between screws, light grey). B) Side view of the head, the truncated cone of the adjustable capillary holder as well as the capillary can be seen (light grey) as well as the angled fan holder.

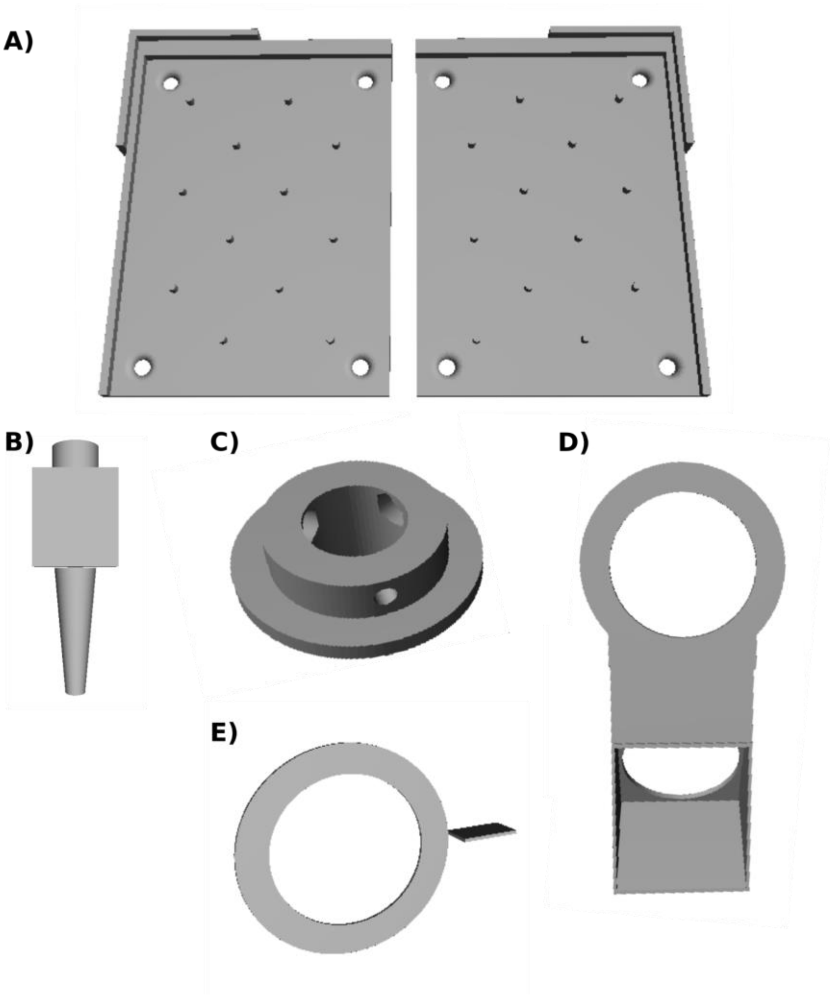

Figure S3: Drawing of the 3D printable parts as uploaded on Zenodo and Thingiverse (see data sharing section in main document). A) Paper holder, designed for one DIN A4 paper (needs to be printed twice). This holder keeps the μPAD in place and elevates it to facilitate drying. Can be installed on the bench surface of the milling machine. B) Spotter head, designed to lead the capillary. The upper part is designed to hold a crimped capillary fitting. C) Inner ring, which holds B) in place. Three holes can be equipped with screws to hold B) in place and to finetune its position. D) Angled fan holder, optional part, can be equipped with a small computer fan to speed up drying of the mobile phase while spotting. Should be installed under C). E) Optional part: a small ring that can be placed beneath part D) and secured using the flap in the small opening of the milling machine holder (see Fig. S2, orange part—gap secured with a screw). This prevents the spotter head from rotating within the milling holder.

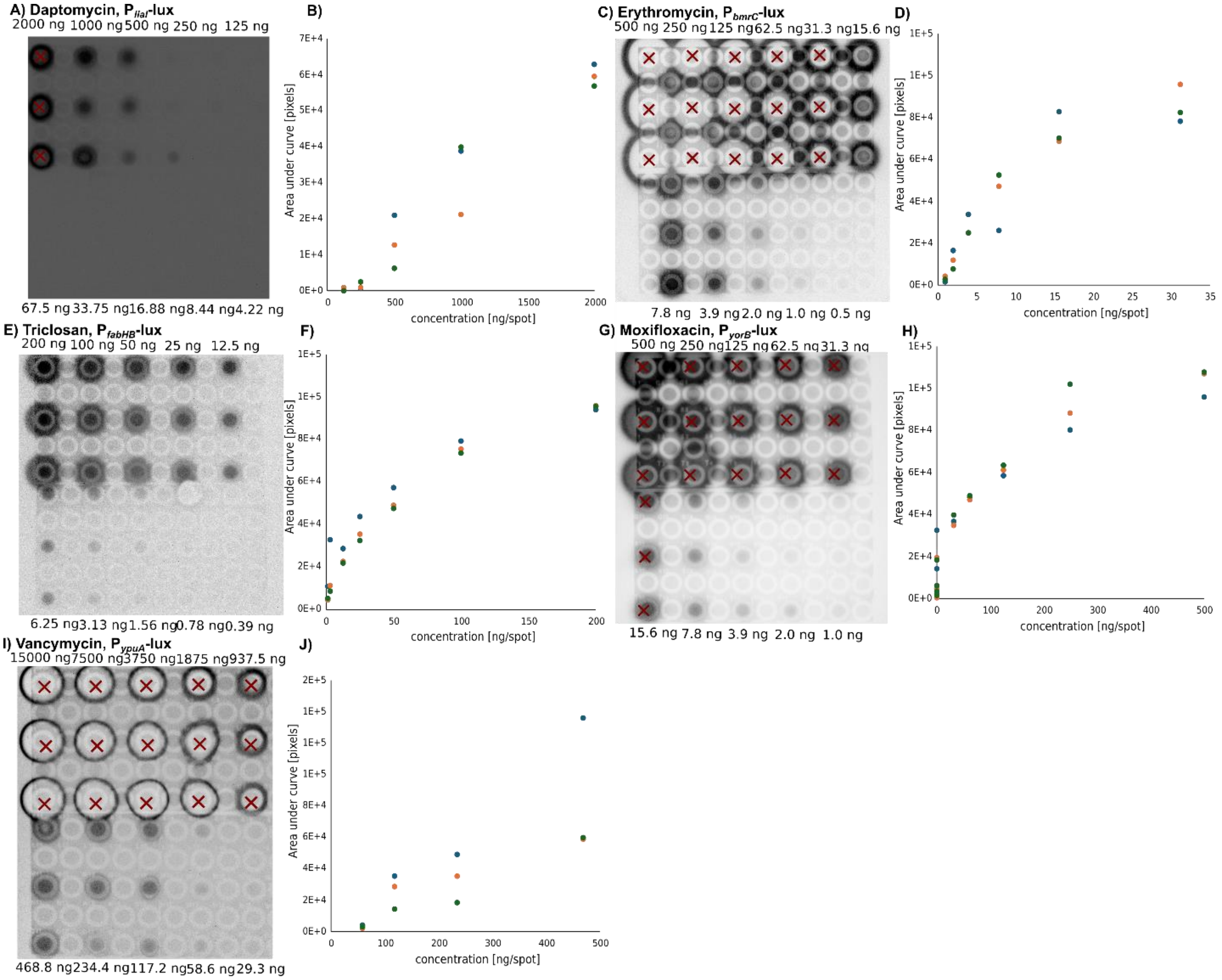

Figure S4: Dilution series of all tested antibiotic standards. The values indicate the concentration of the antibiotic in ng/spot. All concentrations were spotted manually in triplicates. Left side shows the luminescence readout after 3 h. Red crosses indicate spots in which an inhibition zone could be seen by the naked eye after 18 h of incubation. Right side shows the AUC of the spots in pixels plotted against the concentration. (A) and (B): daptomycin, incubated with P_{liaI}-lux reporter strain. (C) and (D): erythromycin, incubated with P_{bmrC}-lux reporter strain. (E) and (F): triclosan, incubated with P_{fabHB}-lux reporter strain. (G) and (H): moxifloxacin, incubated with P_{yorB}-lux reporter strain. (I) and (J): vancomycin, incubated with P_{ypuA}-lux reporter strain.

Of note, it is established that the antibacterial activity of daptomycin is enhanced when Ca^{2+} is added to growth media, per its MoA that involves Ca^{2+} binding. To better simulate „real life conditions“ under which such a specific requirement would not be known when discovering a novel natural product, we refrained in experiment A) from adding Ca^{2+} to the agar for more sensitive daptomycin detection.

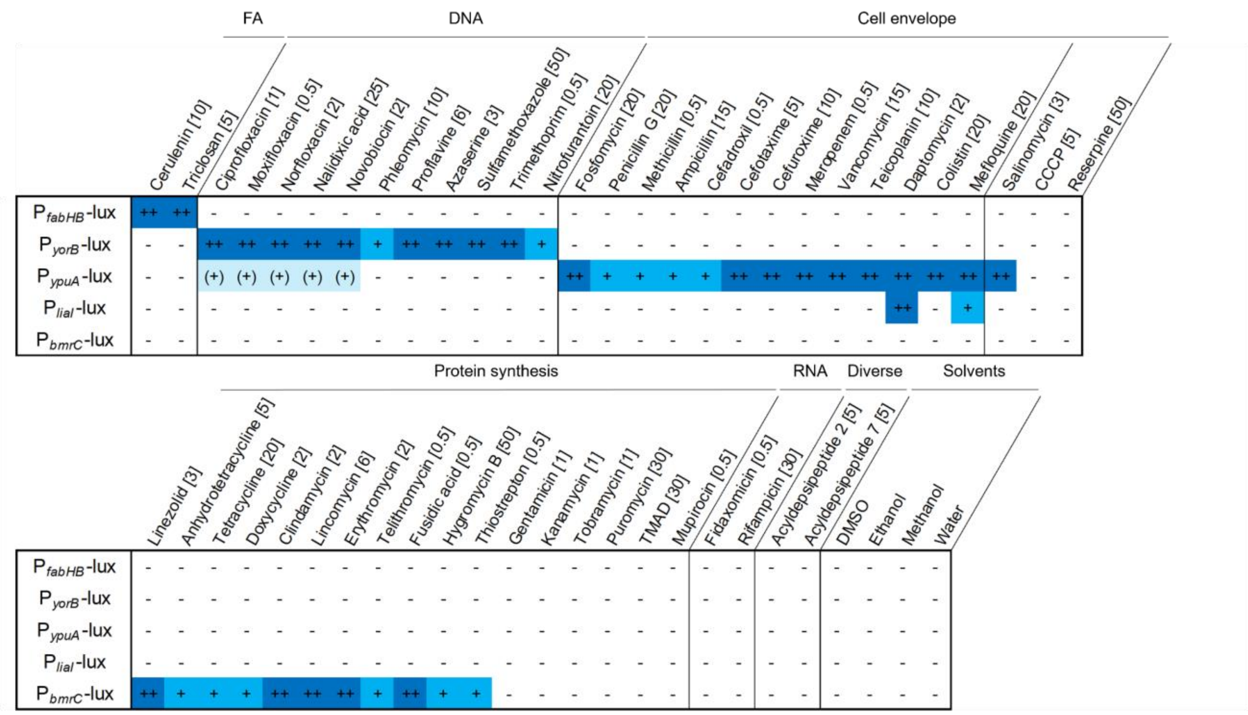

Figure S5A: Agar-based specificity validation. The bioreporter panel was tested in an agar-based setup against 50 reference antibiotics with well-characterized and diverse mechanisms of action, interfering with the depicted major biosynthetic pathways: fatty acid synthesis (FA), DNA and folate synthesis (DNA), cell envelope integrity, protein synthesis, and RNA synthesis (RNA). Likewise, solvent controls (2 µL) were included and showed no effect. Bioreporter induction was quantified by luminescence imaging at 180 min following antibiotic addition and categorized using a four-tier scale: dark blue (++, strong induction), blue (+, good induction), light blue ((+), weak induction) and white (-, no induction). Pure antibacterial agents were directly spotted onto the solidified bioreporter lawn at specified concentrations (µg; amounts denoted in brackets).

	FA		DNA											Cell envelope															
	Cerulenin [10]	Triclosan [5]	Ciprofloxacin [1]	Moxifloxacin [0.5]	Norfloxacin [2]	Nalidixic acid [25]	Novobiocin [2]	Phleomycin [10]	Proflavine [6]	Azaserine [3]	Sulfamethoxazole [50]	Trimethoprim [0.5]	Nitrofurantoin [20]	Fosfomycin [20]	Penicillin G [20]	Methicillin [0.5]	Ampicillin [15]	Cefadroxil [0.5]	Cefotaxime [5]	Cefuroxime [10]	Meropenem [0.5]	Vancomycin [15]	Teicoplanin [10]	Daptomycin [2]	Colistin [20]	Mefloquine [20]	Salinomycin [3]	CCCP [5]	Reserpine [50]
P_{fabHB}-lux	++	++	-	-	-	-	-	-	-	-	-	-	-	-	-	-	-	-	-	-	-	-	-	-	-	-	-	-	-
P_{yorB}-lux	-	-	++	++	++	++	+	++	(+)	++		++	(+)	-	-	-	-	-	-	-	-	-	-	-	-	-	-	-	-
P_{ypuA}-lux	-	-	(+)	(+)	(+)	-	(+)	-	-	-	-	-	-	+	++	+	+	+	++	++	++	+	++	+	++	++	+	-	+
P_{liaI}-lux	-	-	-	-	-	-	-	-	-	-	-	-	-	-	-	-	-	-	-	-	-	++	++	++	-	++	-	-	-
P_{bmrC}-lux	-	-	-	-	-	-	-	-	-	-	-	-	-	-	-	-	-	-	-	-	-	-	-	-	-	-	-	-	-

	Protein synthesis																	RNA		Diverse		Solvents			
	Linezolid [3]	Anhydrotetracycline [5]	Tetracycline [20]	Doxycycline [2]	Clindamycin [2]	Lincomycin [6]	Erythromycin [2]	Telithromycin [0.5]	Fusidic acid [0.5]	Hygromycin B [50]	Thiostrepton [0.5]	Gentamicin [1]	Kanamycin [1]	Tobramycin [1]	Puromycin [30]	TMAD [30]	Mupirocin [0.5]	Fidaxomicin [0.5]	Rifampicin [30]	Acyldepsipeptide 2 [5]	Acyldepsipeptide 7 [5]	DMSO	Ethanol	Methanol	Water
P_{fabHB}-lux	-	-	-	-	-	-	-	-	-	-	-	-	-	-	-	-	-	-	-	-	-	-	-	-	-
P_{yorB}-lux	-	-	-	-	-	-	-	-	-	-	-	-	-	-	-	-	-	-	-	-	-	-	-	-	-
P_{ypuA}-lux	-	-	-	-	-	-	-	-	-	-	-	-	-	-	-	-	-	-	-	-	-	-	-	-	-
P_{liaI}-lux	-	-	-	-	-	-	-	-	-	-	-	-	-	-	-	-	-	-	-	-	-	-	(+)	-	-
P_{bmrC}-lux	+	+	+	+	+	+	++	+	+	+	++	-	-	-	-	-	-	-	-	-	-	-	-	-	-

Figure S5B: Liquid-based specificity validation. The bioreporter panel was tested in an liquid-based setup against 50 reference antibiotics with well-characterized and diverse mechanisms of action, interfering with the depicted major biosynthetic pathways: fatty acid synthesis (FA), DNA and folate synthesis (DNA), cell envelope integrity, protein synthesis, and RNA synthesis (RNA). Likewise, solvent controls (4%) were included and showed no effect. Bioreporter induction was quantified as normalized fold increase relative to untreated controls over a period of 60 min for P_{liaI}-lux, 90 min for P_{fabHB}-lux, 120 min for P_{ypuA}-lux and 180 min for P_{bmrC}-lux and P_{yorB}-lux, based on two biological replicates. Induction levels were categorized using a four-tier scale: dark blue (++, strong induction), blue (+, good induction), light blue ((+), weak induction) and white (-, no induction). Pure antibacterial agents were tested in a two-fold dilution series starting from the indicated concentrations (µg; amounts in brackets).

In general, results from the liquid setup matched the results from the agar-based setup, with the following few exceptions: Lack of P_{yorB}-lux induction by the nucleotide synthesis inhibitor sulfamethoxazole[1] in liquid culture, which may be attributed to the reduced antimicrobial efficacy of sulfamethoxazole observed in this setup. Lack of P_{ypuA}-lux induction by nalidixic acid in liquid culture.[2] The P_{ypuA}-lux bioreporter was induced by the efflux pump inhibitor reserpine[3] in the liquid assay, but not in the agar-based setup. The P_{liaI}-lux bioreporter was induced by the glycopeptides vancomycin and teicoplanin[4] in the liquid assay, but not in the agar-based setup. Exposure to high concentrations of ethanol (≥8% V/V) resulted in a weak induction of P_{liaI}-lux in the liquid setup, consistent with previous reports describing ethanol as a weak P_{liaI} inducer.[5]

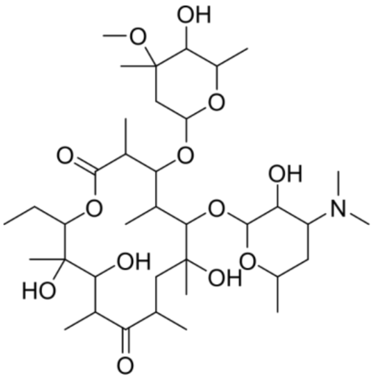

Chemical Formula: $C_{37}H_{67}NO_{13}$
Exact Mass: 733.4612
Erythromycin A

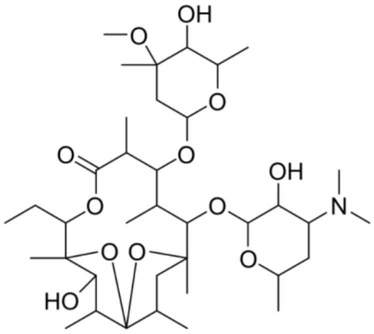

Chemical Formula: $C_{37}H_{65}NO_{12}$
Exact Mass: 715.4507
Anhydroerythromycin A

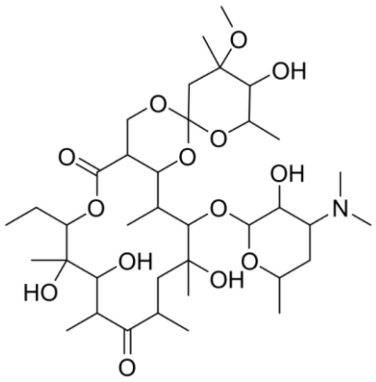

Chemical Formula: $C_{37}H_{65}NO_{14}$
Exact Mass: 747.4405
Erythromycin E

Figure S6: Compounds determined in the crude extract of *S. erythraea* that clustered together in the molecular network as shown in Fig. 4. While erythromycin A gives the main activity peak, anhydroerythromycin A and erythromycin E are present as minor compounds.[6]

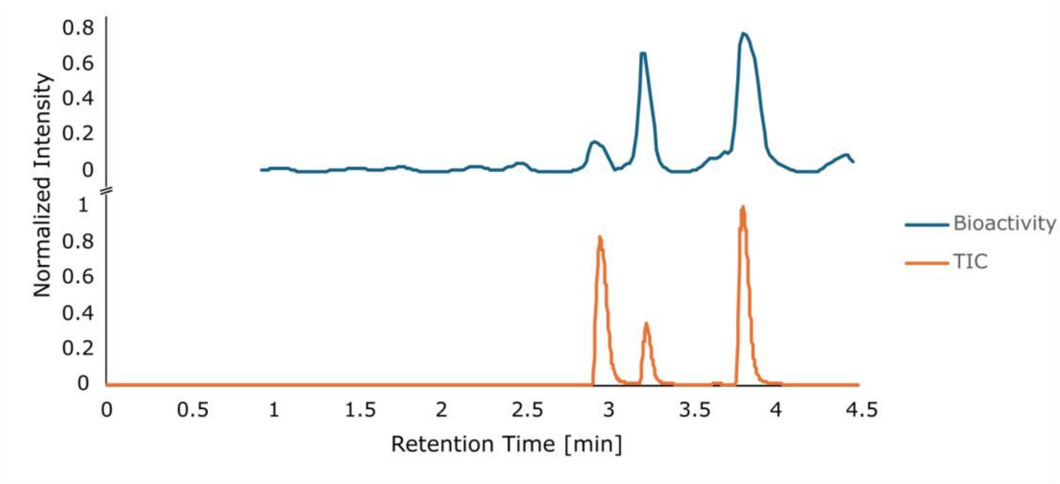

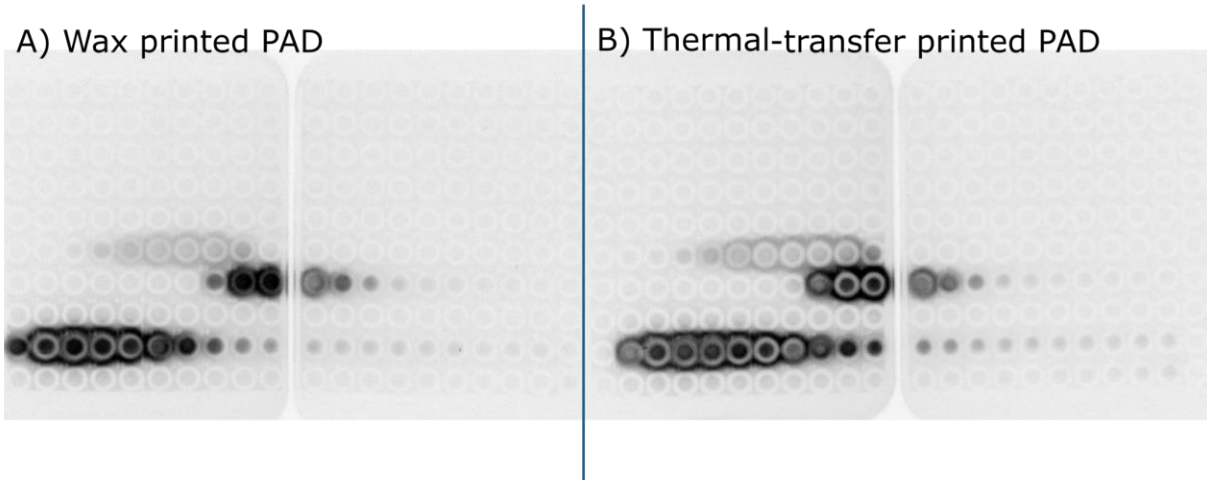

Figure S7: Comparison of PADs printed with wax printer (A) and thermal transfer printer (B). Elution order: Trimethoprim, Ciprofloxacin, Moxifloxacin. See also Fig. 3 and chapter 3.3 in the main document.

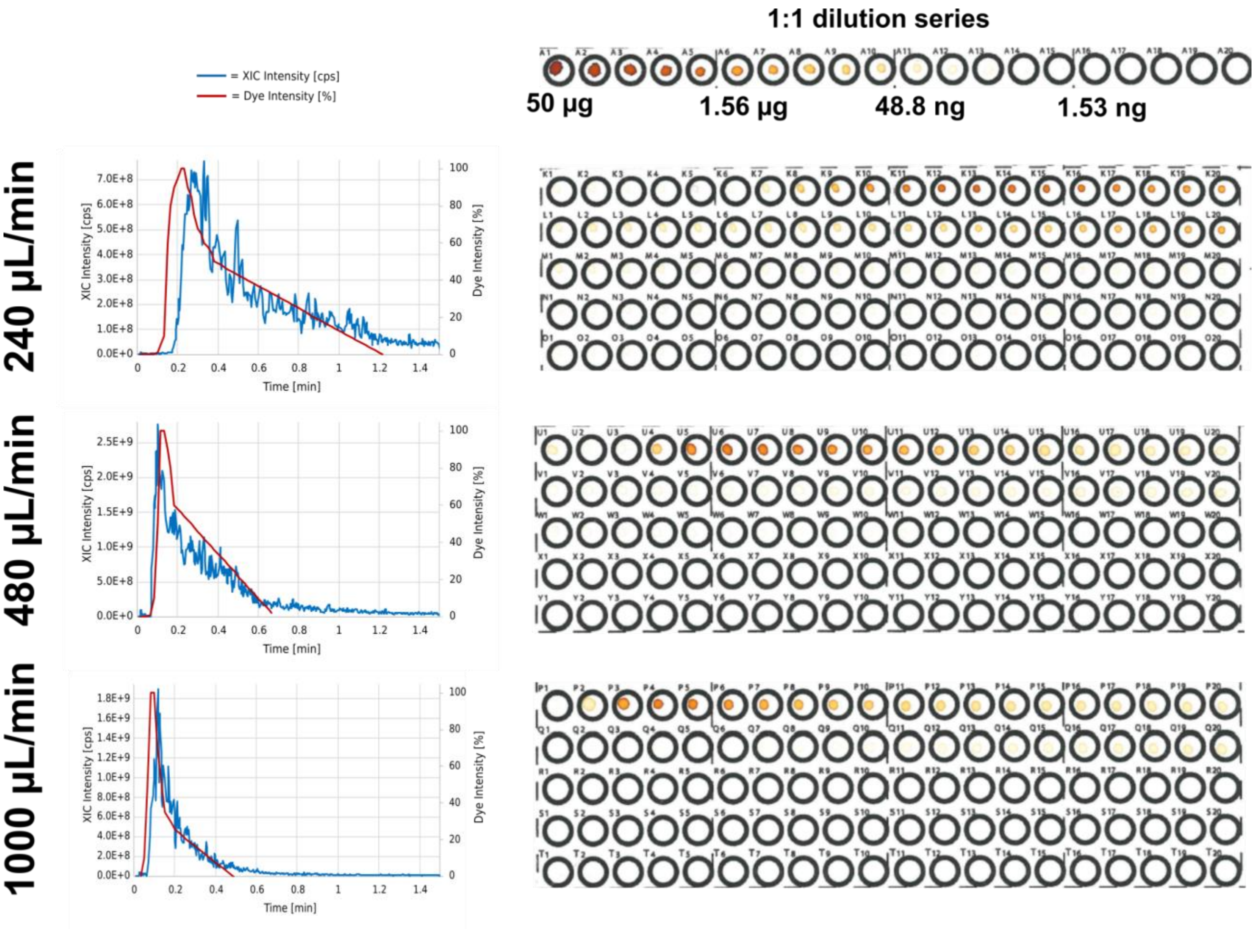

Figure S8: Determination of diffusion effects between MS and Microspotter. To test for possible peak broadening effects and spot-to-spot carryover due to diffusion effects, a sample of a dye (methyl red, 10 mg/mL) was injected in-flow without column at three different flow rates (240, 480, and 1000 µL/min). Upper part: Overview on the spot intensity by manual spotting of a dilution series. Left side: extracted ion chromatograms (XICs) of the sodium adduct of methyl red ($C_{15}H_{15}N_3O_2Na$, *m/z* = 292.1062 ± 10 ppm, blue curves). Curves were smoothed manually applying a 5-point Savitzky-Golay-smoothing. Red curves show the manual readout of the spot intensities Right side: Respective spotted µPADs.

SUPPLEMENTARY METHODS

Cloning of the Lux bioreporters

Bioreporters were constructed by transforming a sporulation-deficient *Bacillus subtilis* 1S34 with a modified version of the integrative reporter vector pBS3Clux.[7,8] This vector harbors an ampicillin resistance gene for selection in *Escherichia coli* on the non-integrative plasmid region, and a chloramphenicol resistance gene situated between the integration sites to facilitate selection in *B. subtilis*. Promoter regions specific to each bioreporter (P_{fabHB}, P_{yorB}, P_{ypuA}, P_{liaI}, P_{bmrC}) were amplified from *B. subtilis* 1S34 genomic DNA and cloned into the pBS3Clux shuttle vector via common restriction-ligation protocols combined with Gibson assembly. Each promoter region was integrated 19 bp upstream of the *Photorhabdus luminescens luxABCDE*-operon, exercising the original mRFP1 red fluorescent protein. Vectors were propagated in *E. coli* XL-10, cultured in lysogeny broth (LB; 1% NaCl, 1% tryptone, 0.5% yeast extract, pH 7.25) containing 100 µg/mL ampicillin, and purified using a GeneJET Plasmid Miniprep Kit (Thermo Scientific). Successful integration of the promoter regions was verified by Sanger sequencing (LGC Genomics GmbH). Naturally competent *B. subtilis* 1S34 cells were prepared by nutrient starvation[9] and transformed with the assembled vectors, which integrated into the chromosomal *sacA* locus. Transformants were selected on 5 µg/mL chloramphenicol, and integration was confirmed via colony PCR. Primers utilized in this study are listed in Table S2. Bioreporter strains were cultivated in LB supplemented with 5 µg/mL chloramphenicol at 37°C and shaking (190 rpm).

Liquid-based bioreporter assay

For each bioreporter a pre-culture was initiated from a glycerol stock and incubated for 18 h, followed by inoculation of a main culture to an initial OD_{600} of 0.05, which was subsequently grown to an OD_{600} of approximately 1.0. Reference antibiotics were prepared in a two-fold serial dilution using a white, clear-flat bottom 96-well plate (Brand) in LB medium for the bioreporter P_{fabHB}-lux, P_{yorB}-lux, P_{ypuA}-lux, P_{liaI}-lux, while BMM[7] was utilized for P_{bmrC}-lux. For solvent controls, 4% of solvent were added to the first well and serially diluted in the same manner. The bioreporters were transferred onto the pre-warmed well plate to a final cell count of 0.95×10^7 CFU/mL, diluted in the respective media (without chloramphenicol). The final assay volume was 120 µL. Luminescence and absorbance measurements at 600 nm were performed at five-minute intervals over an incubation period of 3 h at 37°C using a SPARK multimode microplate reader (Tecan), with the incubation parameters listed in Table S3.

Bacterial growth and bioreporter luminescence were continuously monitored in a microplate reader over a period of 60 min for P_{liaI}-lux, 90 min for P_{fabHB}-lux, 120 min for P_{ypuA}-lux, and 180 min for P_{bmrC}-lux and P_{yorB}-lux following antibiotic addition. These incubation times were selected to ensure specific bioreporter activation, as extended incubation occasionally resulted in nonspecific signals, like in the agar-based assay. Antibiotics were tested in a two-fold dilution series starting at 1x the minimal inhibitory concentration (MIC) or higher. Induction was quantified relative to untreated controls and normalized to both baseline luminescence and the optical density at 600

nm (OD_{600}; as a measure of bacterial growth) of each well. Results from independent biological replicates were reported as mean fold induction values. For assay validation, significant induction thresholds were defined as a ≥500%-fold increase for P_{fabHB}-lux, P_{yorB}-lux, P_{liaI}-lux and P_{bmrC}-lux, and a ≥200%-fold increase for the less responsive promotor P_{ypuA}-lux. Each bioreporter displayed a distinct and selective activation profile that was largely consistent with the results obtained in the agar-based assay. For instances where the two assay formats diverged, see Fig. S5B. Inherent differences in baseline expression and natural differences in promotor regulation preclude direct quantitative comparison between the different bioreporters. Also, an individual bioreporter reacted to different compound classes to a different extent, as a consequence of the diverging mechanisms of action. In summary, each bioreporter strain demonstrated high specificity for its respective mechanisms of action area in both the agar-based and liquid-based bioreporter assay.

Comparison of wax-based and thermal transfer PAD

The wax-based PADs have a long standing history in analytical chemistry.[10] They are extremely cheap and easy to produce, and commercially available printer paper can be used. When a wax printer is at hand, the printing of a PAD only takes seconds, and the design can be freely chosen, which makes it attractive for several applications. As the wax lies on top of the paper, it just has to be heated to ~110°C for about 1 minute, so that the wax melts into the paper and hydrophobizes the paper in the form of the printed wax. Unfortunately, wax printers are, to our knowledge, not produced anymore. As the lack of PADs would be a drawback for this project, we searched for alternative possibilities for the production of PADs. In the publication of Ruiz et al.,[11] we found a comparison of different possibilities to print PADs. We decided to systematically investigate the printing of PADs using a thermal-transfer printer. All chosen timepoints and temperatures for the conditioning of the PADs (90, 105, 120°C and 5, 10, 30 min) were analyzed by spotting 20 µL pure acetonitrile manually into the spots and see if the spots are able to hold the acetonitrile so that it dries inside the ring before running out. The optimum in those conditions was conditioning for 30 minutes at 120°C. A drawback of the printer used in our case was that the printing itself was not always accurate. The first pages often had to be discarded as some circles were not fully printed, and the printer had no proper paper feed; a paper fed askew led to a scheme of circles to-be-spotted in that was not centered and oblique. This could partly be avoided by carefully feeding the paper one by one. Nevertheless, as after conditioning the backing had to be printed to the paper to avoid a migration of the compounds in the agar below, an oblique printing can lead to problems. This printing device might therefore not be as ideal as a wax printer; given the fact that wax printers are outdated and that the thermal transfer printer is cheap and small, we believe that it poses a sufficient replacement. To verify the usability of the PADs obtained from this printer, an antibiotic mixture (ciprofloxacin, trimethoprim, moxifloxacin) was spotted in the same concentration with the same method on both PADs. Both were incubated with P_{yorB}-lux bioreporter to display DNA stress. As seen in Fig. S7, both results are comparable. All antibiotics can be seen in both PADs with comparable intensity. When focusing on the tail of the signals, thermal-transfer PAD might even be superior as it might lower the limit of detection; for all three compounds, more

spots are visible on the thermal-transfer PAD. This indicates that it is a suitable alternative to the wax printed PADs.

Microspotter head fabrication

The 3D printed spotter head consists of four parts (see Figure S2, S3 and CAD files in data sharing section in the main document). Fig. S3B shows the capillary holder, designed as a ring that tapers into a truncated cone to secure the capillary outlet at the bottom. The top end features a crimped capillary fitting connected to a union that can be attached to the outlet capillary of the HPLC. Fig. S3C depicts the outer ring, which includes three holes fitted with screw nuts. These allow screws to be inserted for securing the inner capillary holder. The capillary holder is freely movable along the x- and y-axis by the screws.
Furthermore, a third part was 3D-printed (Fig. S3D), holding a small fan at an angle that allows the fan to blow air over the µPAD to dry it faster while the spotter head is moving. The 5V power supply for the fan is realized by a USB cable.
The fourth part is a small ring that locks the spotter head into the milling machine arm, preventing it from rotating during operation. All of the parts for the spotter head were tailor-made in-house using a 3D-printer with PLA as filament and CAD files are publicly available under the Zenodo repository as well as via Thingiverse (see data sharing section in main document).

Spot-to-spot carryover

Carryover of eluate from one spot to the next on the µPAD can be a drawback for the determination of bioactivity based on retention time. Therefore, spot-to-spot carryover should be critically assigned and avoided if possible. As the capillary tip on the Microspotter in our setup does not come into contact with the µPAD, a contamination or carryover due to contact is unlikely. The hanging drop is transferred by capillary forces onto the µPAD and should lead to a complete detachment once the spotter head is moving back up before continuing to the next spot. However, to experimentally evaluate this, we performed flow injections at different flow rates to compare detector responses of the MS and compound deposition on the µPAD. To do so, we used methyl red (10 mg/mL) as a test compound, as it allows for easy optical evaluation of compound deposition. Three different flow rates were evaluated (240, 480, 1000 µL/min), spotting frequency was 1 Hz. As both detectors reacted to the flow rate resp. peak width changes comparably (see Fig. S8), we argue that the prolonged signal for broader peaks at low flow rates on the µPAD arises not from a significant spot-to-spot carryover but rather from diffusion effects within the capillary. This is also supported by the flow rate dependence of the MS signal. At higher flow rates, the peak is significantly sharper in both the MS trace and the µPAD. In accordance to Fick's first law of diffusion, broadening of the injection band can be described by $\sigma^2 = 2Dt$ (with σ^2 being the injection band variance, D being the diffusion coefficient and t the residence time in the capillary), as the injection band moves in open tubular capillaries. This equation shows a time dependence, which can directly be translated into a flow rate dependence. No column was used

in this experiment, hence refocusing of the injection band on the column head did not take place. Post-column diffusion effects are typically smaller than the diffusion seen in this experiment. For flow rates used in our experiments (1 mL/min), post-column diffusion is low, since the residence time in the capillary is short. While spot-to-spot carryover cannot fully be ruled out, the result of this experiment shows that the carryover can be expected to be low or insignificant when working at a flow rate of 1 mL/min.

Supplementary References

1. Hitchings, G. H. Mechanism of Action of Trimethoprim-Sulfamethoxazole--I. *J. Infect. Dis.* **128**, S433–S436 (1973).
2. Anderson, V. E., Zaniewski, R. P., Kaczmarek, F. S., Gootz, T. D. & Osheroff, N. Quinolones Inhibit DNA Religation Mediated by Staphylococcus aureus Topoisomerase IV. *J. Biol. Chem.* **274**, 35927–35932 (1999).
3. Li, G. *et al.* Antimycobacterial activity of five efflux pump inhibitors against Mycobacterium tuberculosis clinical isolates. *J. Antibiot. (Tokyo)* **69**, 173–175 (2016).
4. Reynolds, P. E. Structure, biochemistry and mechanism of action of glycopeptide antibiotics. *Eur. J. Clin. Microbiol. Infect. Dis.* **8**, 943–950 (1989).
5. Mascher, T., Zimmer, S. L., Smith, T.-A. & Helmann, J. D. Antibiotic-Inducible Promoter Regulated by the Cell Envelope Stress-Sensing Two-Component System LiaRS of *Bacillus subtilis*. *Antimicrob. Agents Chemother.* **48**, 2888–2896 (2004).
6. Deubel, A., Fandiño, A. S., Sörgel, F. & Holzgrabe, U. Determination of erythromycin and related substances in commercial samples using liquid chromatography/ion trap mass spectrometry. *J. Chromatogr. A* **1136**, 39–47 (2006).
7. Stülke, J. & Hanschke, R. Temporal activation of β-glucanase synthesis in Bacillus subtilis is mediated by the GTP pool. *J. Gen. Microbiol.* **139**, 2041–2045 (1993).
8. Radeck, J. *et al.* The Bacillus BioBrick Box: generation and evaluation of essential genetic building blocks for standardized work with Bacillus subtilis. *J. Biol. Eng.* **7**, 29 (2013).
9. Newcastle iGEM Project Wiki (2009). Transforming into Bacillus subtilis 168. https://2009.igem.org/Team:Newcastle/Project/Labwork/OurProtocols/TransformBac, accessed 2025-07-08.
10. Martinez, A. W., Phillips, S. T., Butte, M. J. & Whitesides, G. M. Patterned Paper as a Platform for Inexpensive, Low-Volume, Portable Bioassays. *Angew. Chem. Int. Ed.* **46**, 1318–1320 (2007).
11. Ruiz, R. A., Gonzalez, J. L., Vazquez-Alvarado, M., Martinez, N. W. & Martinez, A. W. Beyond Wax Printing: Fabrication of Paper-Based Microfluidic Devices Using a Thermal Transfer Printer. *Anal. Chem.* **94**, 8833–8837 (2022).
